# Spectral CT electron density for evaluation of lumbar intervertebral disk degeneration

**DOI:** 10.1371/journal.pone.0357658

**Published:** 2026-09-01

**Authors:** Masanori Nakajo, Tsubasa Nakano, Tomohito Hasegawa, Kiyohisa Kamimura, Koji Takumi, Hiroaki Nagano, Masatoyo Nakajo, Hiroyuki Tominaga, Mutsukazu Hayashi, Takashi Iwanaga, Fumiko Kanzaki, Eran Langzam, Graeme Campbell, Ko Higuchi, Takashi Yoshiura

**Affiliations:** 1 Department of Radiology, Kagoshima University Graduate School of Medical and Dental Sciences, Kagoshima, Japan; 2 Department of Orthopaedic Surgery, Kagoshima University Graduate School of Medical and Dental Sciences, Kagoshima, Japan; 3 Department of Radiological Technology, Kagoshima University Hospital, Kagoshima, Japan; 4 CT Clinical Science, Philips Healthcare, Haifa, Israel; 5 CT Clinical Science, Philips GmbH Market DACH, Hamburg, Germany; 6 CT Clinical Science, Philips Japan, Tokyo, Japan; IPM: Institute for Research in Fundamental Sciences, IRAN, ISLAMIC REPUBLIC OF

## Abstract

**Objective:**

This study aimed to investigate the ability of spectral CT parameters to evaluate lumbar intervertebral disk degeneration (LIDD).

**Summary of background data:**

LIDD is the most prevalent cause of low back pain. However, the usefulness of spectral CT for LIDD evaluation remains unclear.

**Methods:**

This retrospective study included consecutive patients who underwent spectral CT and Magnetic resonance imaging for spinal disease examination from February 2018 to June 2021 on a single center. The conventional polyenergetic 120-kVp computed tomography value (CT_conv_), electron density (ED), and effective atomic number (Z_eff_) were measured in the annulus fibrosus (AF) and nucleus pulposus (NP) in each disk. Additionally, differences in each spectral CT parameter were obtained between the anterior AF and NP and between the posterior AF and NP. These parametric values were correlated with modified Pfirrmann grade (mPG) using Spearman’s rank correlation coefficients. The receiver operating characteristic curve analysis was used to assess diagnostic performances for differentiating between normal and degenerative disks.

**Results:**

Three hundred thirty-eight lumbar intervertebral disks of 100 patients were analyzed. The CT_conv_ and ED were negatively correlated with mPG in AF (ρ = −0.29 to −0.36) and positively correlated with mPG in NP (ρ = 0.16–0.45), whereas Z_eff_ in NP was negatively correlated with mPG (ρ = −0.29 to −0.38). The ED differences between the anterior AF and NP and between the posterior AF and NP were strongly correlated with mPG (ρ = −0.71 and −0.64, respectively) and demonstrated high diagnostic performances in differentiating between normal and degenerative disks (area under the receiver operating characteristic curve values = 0.92 and 0.88, respectively).

**Conclusion:**

Spectral CT parameters, particularly ED, can diagnose lumbar intervertebral disk degeneration.

## Introduction

Lumbar intervertebral disk degeneration is the most prevalent cause of low back pain [1,2], and it causes herniated disk disease, lumbar degenerative spondylosis, and lumbar spinal canal stenosis [3].

The intervertebral disk is centrally and peripherally composed of the nucleus pulposus (NP) and the annulus fibrosus (AF), respectively. The AF consists of a fibrocartilaginous structure that primarily contains type I collagen, whereas the NP is rich in water and proteoglycans in a loose network of type II collagen [4]. Proteoglycans in disk degeneration, which function to bind water, decrease, thereby causing dehydration and relatively increased type I collagen within the NP, and tears and degenerate cysts of AF [5]. Lumbar intervertebral disk degeneration evaluation is conventionally performed using Magnetic resonance imaging (MRI) [1,2,5–7]. Pfirrmann grade and modified Pfirrmann grade (mPG) based on T2-weighted images are predominantly used for grading degenerative disk disease [6,7]. Thus, MRI is useful for intervertebral disk degeneration evaluation. However, MRI is unavailable for patients with contraindicated metal implantation or claustrophobia. Moreover, MRI may not be an optimal tool for diagnosing other causative diseases of lower back pain than spinal diseases such as urinary tract stones, aortic dissection, etc. Computed tomography (CT) effectively evaluated these diseases, but its usefulness for lumbar intervertebral disk degeneration evaluation has not been established.

Spectral CT imaging encompasses a unique generation of CT systems that utilize energy-dependent information, with dual-energy CT (DECT) representing its first generation, which can be implemented through various technical approaches including dual-source CT, rapid kVp-switching, and dual-layer spectral detector CT (DLCT) [8]. DECT analyzes data of Compton scattering and the photoelectric effect of X–rays of two energies using various algorithms. DECT studies of intervertebral disk diseases have been reported in recent years, specifically including virtual non-calcium imaging for disk herniation and degeneration [9–13], water imaging for disk degeneration [14], collagen mapping for disk degeneration [15–17], and electron density (ED) imaging for disk herniation [12,13]. ED and effective atomic number (Z_eff_) are recently available as DECT derived parameters [18]. DECT enable more accurate measurements of ED and Z_eff_ through improved dose distribution accuracy, which were difficult to obtain with conventional single-energy CT [19]. While these measurements have been primarily investigated for improving dose calculation accuracy in radiation treatment planning [20], their utility in diagnostic imaging has remained underexplored. Recently, however, the diagnostic value of ED for evaluating disk herniation has been reported [12,13]. To the best of our knowledge, the use of ED and Z_eff_ images for lumbar intervertebral disk degeneration evaluation remains unknown. Therefore, this study aimed to investigate whether spectral CT-derived ED and Zeff can accurately differentiate varying degrees of lumbar disk degeneration.

## Materials and methods

### Patient population

**Fig 1:**
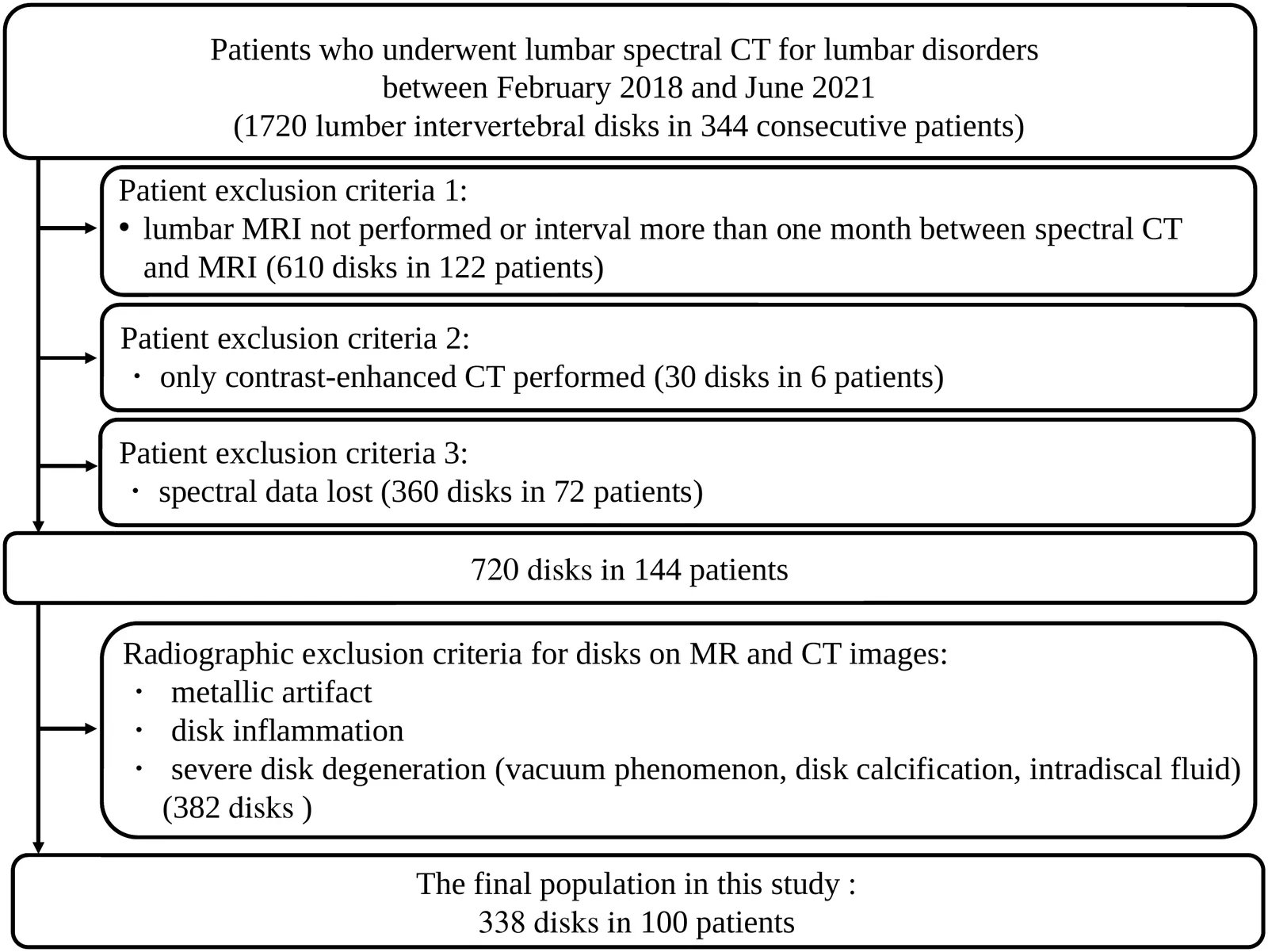
Inclusion flowchart of study participants and lumbar intervertebral disks.

The institutional review board approved this retrospective study under protocol number 200284 and waived the need to obtain informed consent because of the retrospective study design. The data used in this study were accessed on 19 March 2021 for research purposes. We accessed the data that had been appropriately anonymized to ensure the privacy of individual participants. The inclusion criterion was consecutive patients with suspected lumbar disorders who underwent lumbar spectral CT from February 2018 to June 2021. The exclusion criteria were *(a)* no lumbar MRI available, or an interval of more than one month between spectral CT and MRI examinations; *(b)* only contrast-enhanced CT scan available; *(c)* missing spectral data of spectral CT. Intervertebral disks with metallic artifacts, disk inflammation, and severe disk degeneration findings, including vacuum phenomenon, disk calcification, intradiscal fluid, and severe disk height reduction that precluded region of interest (ROI) placement as well as herniated disks, were further excluded. Consequently, the final population comprised 338 lumbar intervertebral disks of 100 patients. (Fig 1). We used the STARD reporting guideline [21] to draft this manuscript, and the STARD reporting checklist [22] when editing, included in S5 Checklist.

### Spectral CT imaging protocols

The CT scanner involved a 64 multidetector row DLCT system (IQon Spectral CT; Philips Healthcare) which is equipped with a dual-layer spectral detector and a single X–ray source constantly enabling the acquisition of high and low-energy projection data and retrospective dual-energy data analysis. The imaging parameters were tube voltage of 120 kVp, gantry rotation time of 0.4 s, effective tube current–exposure time product of 140 mAs with automodulation, pitch of 0.703, and detector-row configuration of 64 × 0.625 mm. Images of the conventional polyenergetic 120-kVp computed tomography value (CT_conv_), ED, and Z_eff_ were generated using a dedicated workstation (IntelliSpace Portal; Philips Healthcare). ED denotes the number of electrons per unit volume and is presented as a relative value to the ED of water in percent (%EDW) [23]. Z_eff_ indicates the effective atomic number when the compound consists of several elements and is replaced by one of the elements [24].

### MRI protocols

All MRI scans were conducted using 3.0-T systems (Achieve dStream 3.0 T; Philips Healthcare, Best, the Netherlands and Prisma; Siemens, Erlangen, Germany). S1 Table summarizes the sagittal T2-weighted imaging protocols.

### Image analysis

Two independent radiologists (17 and 8 years of experience in CT and MRI interpretation centered on neuroradiology) evaluated images from spectral CT and MRI. The readers were blinded to the clinical information of the patients and individually evaluated spectral CT and MRI data at 1-month intervals. They evaluated lumbar intervertebral disk degeneration according to the mPG [10]. For cases with discordant grades, the mPG was determined by the consensus of the two radiologists. Each intervertebral disk was further categorized as normal (mPG1 and 2) or degenerated (mPG3–8) [24–26].

**Fig 2:**
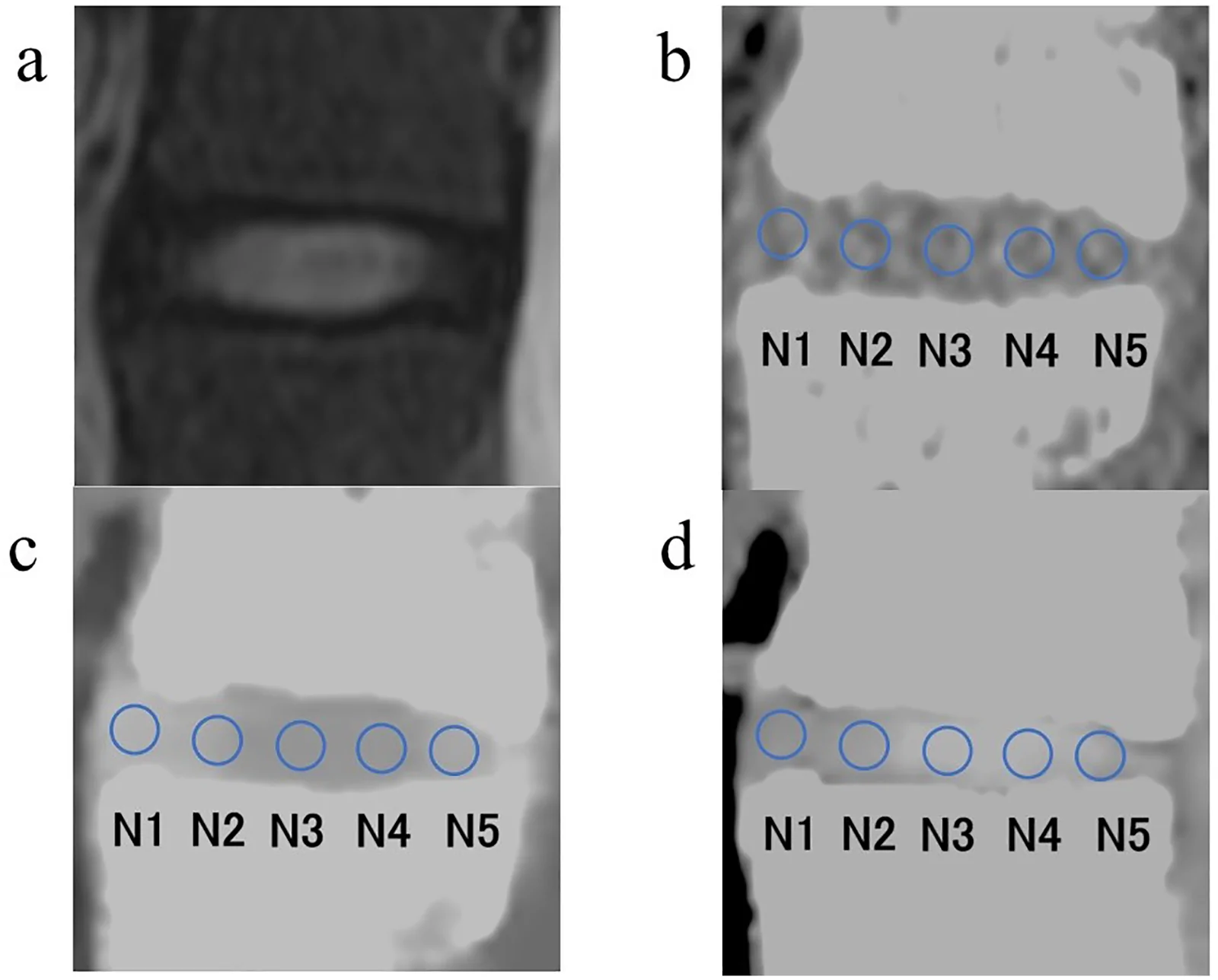
ROIs in each spectral CT image. (a) T2-weighted, (b) CT_conv_, (c) ED, and (d) Z_eff_. The ROIs are labeled as N1 − N5 from anterior to posterior, with N1 and N5 in the AF and N2 − 4 in the NP.

Five ROIs (10 mm^2^) were drawn in each disk on the midsagittal plane, labeled as N1–N5 from anterior to posterior—N1 and N5 in AF and N2–N4 in NP (Fig 2).

ROIs were set on CT_conv_ images and then duplicated across ED and Z_eff_ images. The mean CT_conv_, ED, and Z_eff_ were measured for each ROI using a dedicated workstation (IntelliSpace Portal; Philips Healthcare). Furthermore, differences between anterior AF and NP, i.e., Δ(N1 − N3) and between posterior AF and NP, i.e., Δ(N5 − N3) were obtained for all parameters.

### Statistical analysis

The software packages Statistical Package for the Social Sciences version 28.0.1.0 (SPSS, Chicago, IL, USA) and MedCalc (ver. 22.013; Mariakerke, Belgium) were used for statistical analyses. Kolmogorov–Smirnov test was used to assess data normality. Inter-observer agreement was evaluated using the intraclass correlation coefficient (ICC) for parameters with normal distribution and the weighted kappa analysis for parameters with non-normal distribution, respectively (ICC and κ-values of.00–0.20, 0.21–0.40, 0.41–0.60, 0.61–0.80, and 0.81–1.00 indicate poor, fair, moderate, substantial, and almost perfect agreement, respectively).

The ROI measurements from the two readers were averaged for further analyses. The ROI mean values for N1 to N5, Δ(N1 − N3), and Δ(N5 − N3) of all parameters were correlated with mPG using the Spearman’s rank correlation coefficient (|ρ| of 0.00–0.19, 0.20–0.39, 0.40–0.59, 0.60–0.79, and 0.80–1.00 indicate very weak, weak, moderate, strong, and very strong correlations, respectively). The independent samples T-test and the Mann–Whitney U test were used for parameters with normal distribution and those with non-normal distribution, respectively, to compare all parameters between normal (mPG1 and 2) and degenerated disks (mPG3–8). The receiver operating characteristic curve analysis was used to assess its diagnostic ability in differentiating normal and degenerated disks on all parameters. The sensitivity, specificity, and accuracy for differentiating degenerated disks (mPG3–8) from normal disks (mPG1 and 2) were calculated using a threshold criterion that was identified by the largest Youden index [27]. The area under the receiver operating characteristic curve (AUC) values of all parameters for the five ROIs, Δ(N1 − N3), and Δ(N5 − N3) were compared using the DeLong method [28]. A *P* value of < 0.05 indicates a significant difference.

## Results

### Patient characteristics

The final population included 338 lumbar intervertebral disks of 100 patients (mean age, 58.5 ± 20.0 years; range, 10–89 years, 55 men and 45 women) (Table 1).

**Table 1:** Participant characteristics.

Characteristic	Value
Number of patients	100
Number of lumber intervertebral disks	338
L1/2	74
L2/3	73
L3/4	62
L4/5	56
L5/S1	73
Mean age (y)*	58.5 ± 20.0
Sex (men, women)	(55, 45)
Clinical diagnoses	
Lumbar spinal canal stenosis	26
Lumbar vertebral fracture	12
Scoliosis	12
Spinal schwannoma	10
Lumbar disk herniation	9
Lumbar spondylolysis	4
Spinal dysraphism	4
Pyogenic spondylitis	3
Ossification of the yellow ligament	2
Perineural cyst	2
Spinal arteriovenous fistula	2
Spinal meningioma	2
Spinal vertebral metastasis	2
Synovial cyst	2
Adhesive arachnoiditis	1
Spinal cerebrospinal fluid leakage	1
Lumbar kyphosis	1
Spinal cavernous hemangioma	1
Spinal choroid plexus papilloma	1
Spinal meningocele	1
Spinal tuberculosis	1
Spondylitis	1

Unless otherwise indicated, data are number of patients. *Age is presented as the mean ± standard deviation.

### The mPG of lumbar intervertebral disks

**Fig 3:**
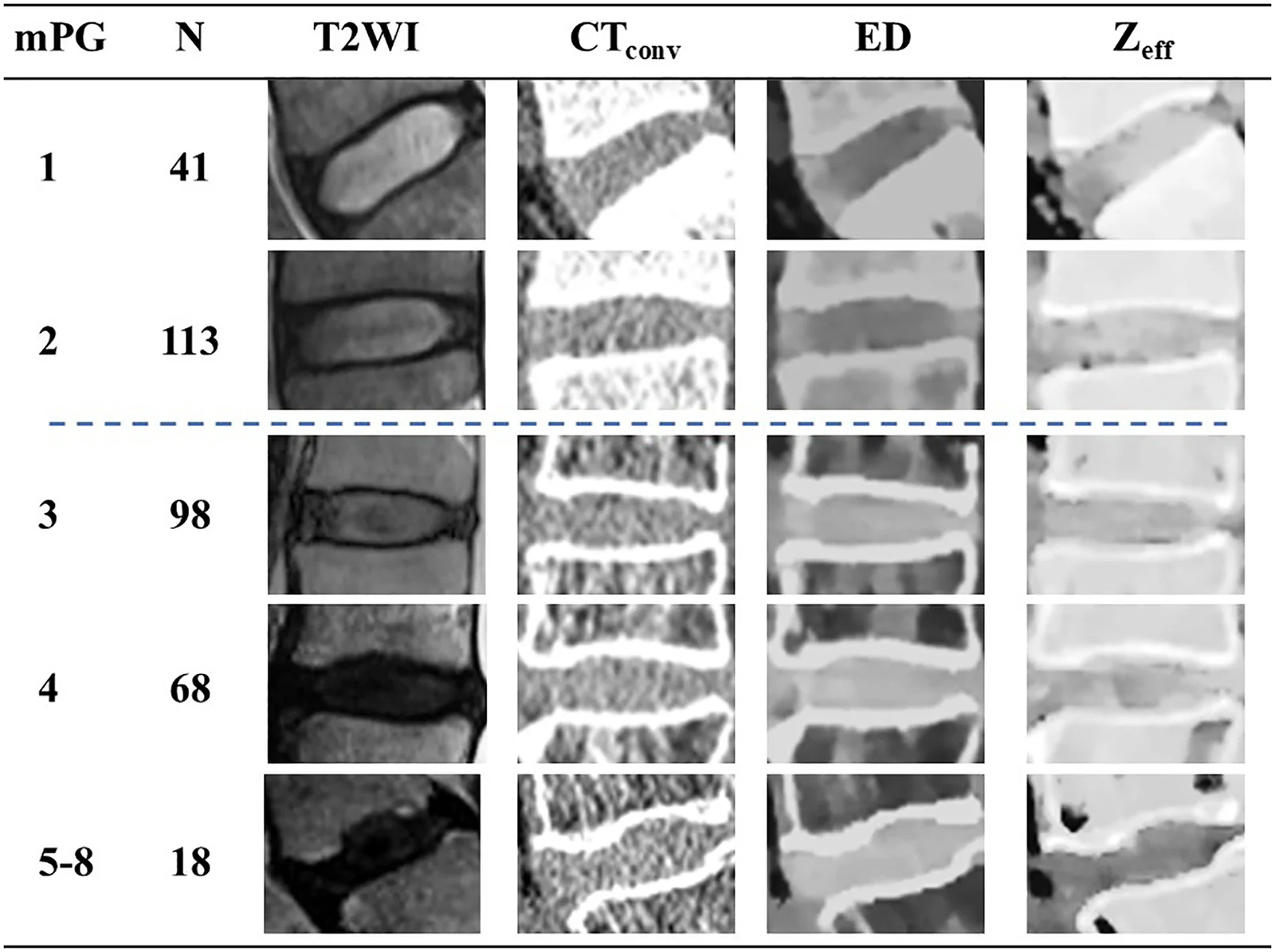
The mPG classification of 338 lumbar intervertebral disks.

The 338 lumbar intervertebral disks were classified following mPG with normal disks in 154 (mPG 1, n = 41; mPG 2, n = 113) and degenerated disks in 184 (mPG 3, n = 98; mPG 4, n = 68; mPG 5–7, n = 18; mPG 8, n = 0). Fig 3 shows representative T2-weighted MRI and spectral CT parametric images for each mPG.

### Inter-observer agreements of mPG and spectral CT parameters

The inter-observer agreement for the mPG was substantial (κ = 0.80, 95% confidence interval [CI]: 0.76–0.84). The inter-observer agreement for the CT_conv_ was moderate (ICC = 0.56, 95% CI: 0.52–0.59), whereas those for ED and Z_eff_ were almost perfect (ICC = 0.86, 95% CI: 0.85–0.87), and substantial (ICC = 0.73, 95% CI: 0.71–0.75), respectively.

### Correlation between spectral CT parameters and lumbar intervertebral disk degeneration

**Fig 4:**
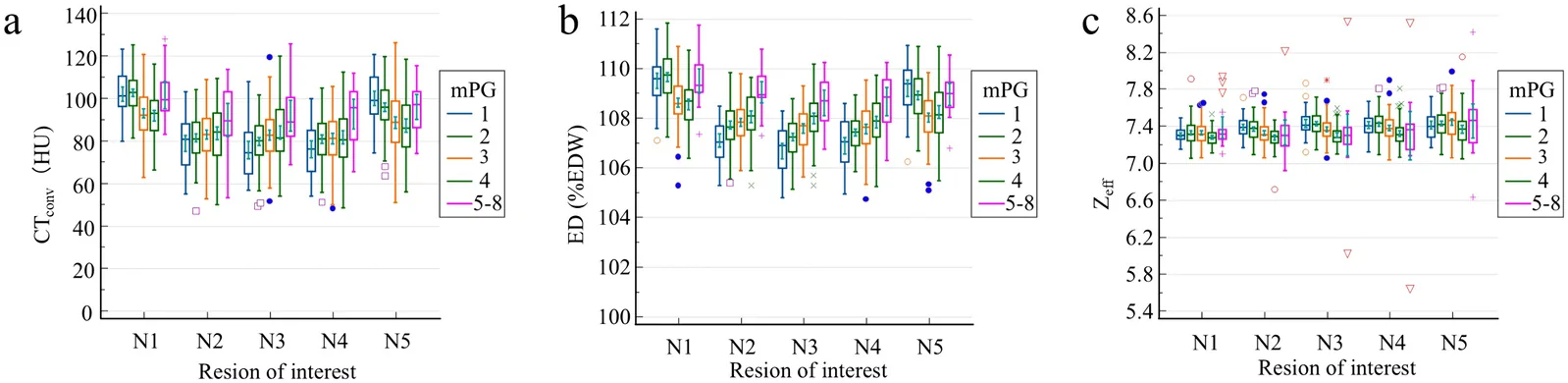
Box plots of mean CT_conv,_ ED, and Z_eff_ values in 5 ROIs following the mPG. (a) CT_conv_ values in the NP (N2 − 4) increase, whereas those in the AF (N1, and 5) decrease as mPG increases with disk degeneration progression. (b) Similarly, ED values in NP increase, and those in AF decrease with increasing mPG. (c) The Z_eff_ values in NP decrease with increasing mPG. However, the Z_eff_ values in AF demonstrate no obvious change with mPG.

Fig 4 shows the states in mean CT_conv_, ED, and Z_eff_ N1–5 associated with the mPGs of lumbar intervertebral disk degeneration.

Table 2 summarizes the correlations between spectral CT parameters and mPGs in AF (N1 and N5) and NP (N2–4) and their differences between AF and NP.

**Table 2:** Correlations between spectral CT parameters and mPG in AF, NP, and their differences.

ROIs	CT_conv_		ED		Z_eff_	
	ρ	*P* value	ρ	*P* value	ρ	*P* value
N1	−0.33	<0.001	−0.36	<0.001	−0.08	0.151
N2	0.16	0.004	0.34	<0.001	−0.30	<0.001
N3	0.24	<0.001	0.45	<0.001	−0.38	<0.001
N4	0.16	0.003	0.31	<0.001	−0.29	<0.001
N5	−0.29	<0.001	−0.29	<0.001	−0.08	0.166
Δ(N1 − N3)	−0.52	<0.001	−0.71	<0.001	0.34	<0.001
Δ(N5 − N3)	−0.46	<0.001	−0.64	<0.001	0.25	<0.001

AF: Annulus fibrosus; CT_conv_: Conventional polyenergetic 120-kVp computed tomography; ED: Electron density; mPG: Modified pfirrmann grade; NP: Nucleus pulposus; Δ(N1 − N3): Difference between anterior AF and NP; Δ(N5 − N3): Difference between posterior AF and NP; ROIs: Regions of interest; Z_eff_: Effective atomic number.

The mean CT_conv_ values in NP were positively very weakly or weakly correlated with mPG (ρ = 0.16–0.24, *P* < 0.001, or *=* 0.004), whereas those in AF were negatively weakly correlated with mPG (ρ = −0.33, and −0.29, each *P* < 0.001). The mean ED values in NP were positively weakly or moderately correlated with mPG (ρ = 0.31–0.45, each *P* < 0.001), whereas those in AF were negatively weakly correlated with mPG (ρ = −0.36, and −0.29, each *P* < 0.001). The mean Z_eff_ values in NP were negatively weakly correlated with mPG (ρ = −0.29 – −0.38, each *P* < 0.001), but we revealed no evidence of a correlation between the Z_eff_ values in AF and mPG (*P* = 0.151, and 0.166). Δ(N1 − N3) and Δ(N5 − N3) of CT_conv_ were negatively moderately correlated with mPG (ρ = −0.52, and −0.46, each *P* < 0.001), whereas those of ED were negatively strongly correlated with mPG (ρ = −0.71, and −0.64, each *P* < 0.001) and those of Z_eff_ were positively weakly correlated with mPG (ρ = 0.34, and 0.25, each *P* < 0.001).

### Comparison of spectral CT parameters between normal and degenerated disks

**Fig 5:**
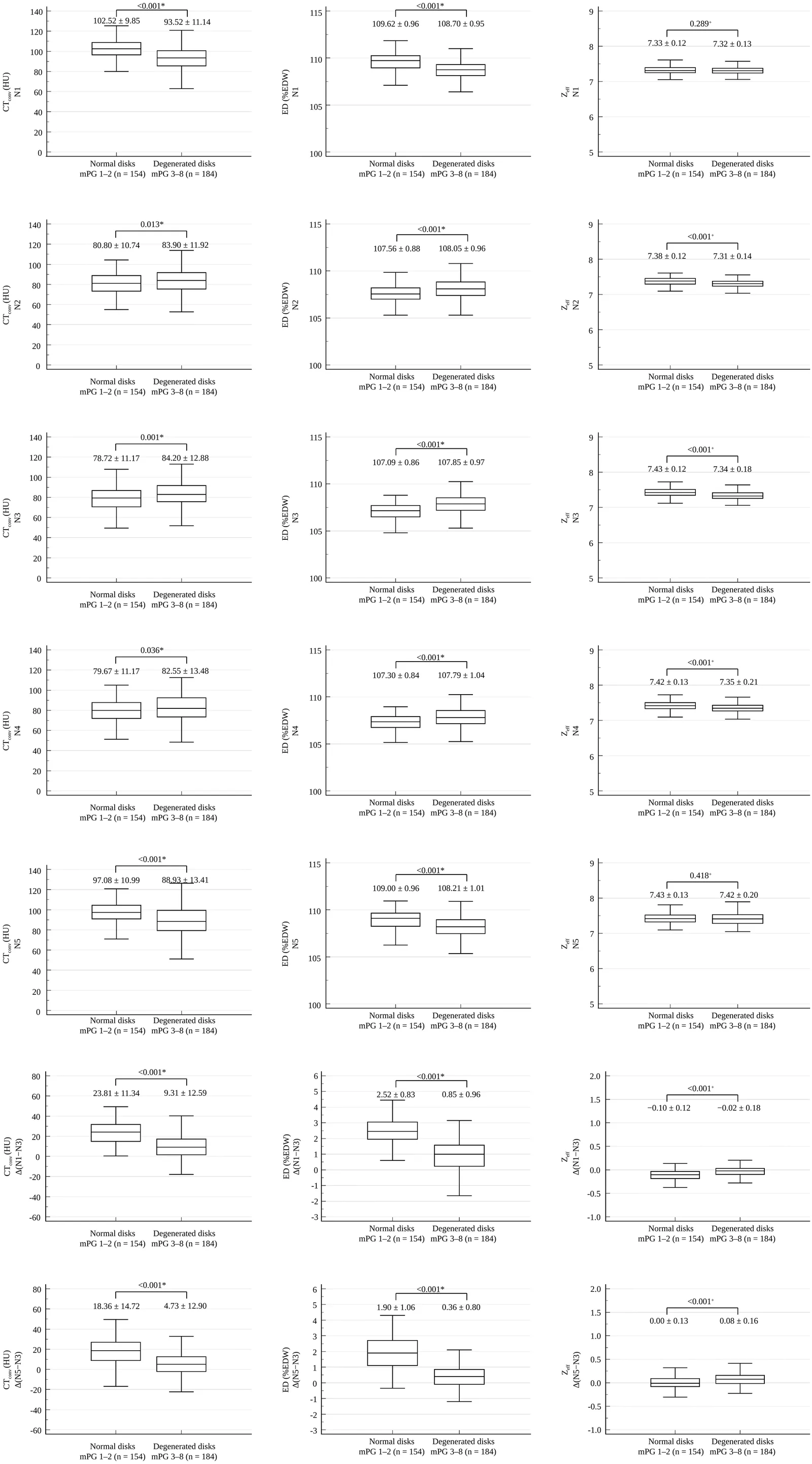
Comparisons of spectral CT parameters in AF, NP, and their differences between normal and degenerated disks. Mean data are ± standard deviations, with ranges in parentheses. AF: Annulus fibrosus; CT_conv_: Conventional polyenergetic 120-kVp computed tomography value; ED: Electron density; %EDW: A relative value to the ED of water in percent; mPG: Modified pfirrmann grade; NP: Nucleus pulposus; Δ(N1 − N3): Difference between anterior AF and NP; Δ(N5 − N3): Difference between posterior AF and NP; Z_eff_: Effective atomic number. * independent samples T-test, ^+^ Mann–Whitney U test.

Fig 5 shows comparisons of spectral CT parameters in NP and AF between normal and degenerated disks. The CT_conv_ and ED values in NP were higher in degenerated disks (82.55–84.20 HU, 107.79–108.05%EDW) than in normal disks (78.72–80.80 HU, 107.09–107.56%EDW) (*P* < 0.001–0.004), whereas Z_eff_ was lower in degenerated disks (7.31–7.35) than in normal disks (7.38–7.43) (each *P* < 0.001). S2 Table summarizes complete crosstabulation of spectral CT parameters between normal and degenerated intervertebral disks.

The CT_conv_ and ED values in AF were lower in degenerated disks (93.52 and 88.93 HU, 108.70 and 108.21%EDW) than in normal disks (102.52 and 97.08HU, 109.00 and 109.62%EDW) (each *P* < 0.001), whereas we found no evidence of a difference in Z_eff_ between normal and degenerated disks. Δ(N1 − N3) and Δ(N5 − N3) of CT_conv_ and ED were lower in degenerative disks (9.31 and 4.73 HU, 0.85 and 0.36%EDW) than in normal disks (23.81 and 18.36 HU, 2.52 and 1.90%EDW) (each *P* < 0.001), whereas those of Z_eff_ were higher in degenerated disks (−0.02 and 0.08) than in normal disks (−0.10 and 0.00) (each *P* < 0.001).

### Diagnostic performances of spectral CT parameters in differentiating degenerated disks from normal disks

The AUC values of CT_conv_ for differentiating degenerated disks from normal disks were 0.73 and 0.68 in AF and 0.56–0.62 in NP, whereas 0.76 and 0.71 in AF and 0.64–0.72 in NP for ED, and 0.65–0.71 in NP for Z_eff_, respectively (Table 3).

**Table 3:** Diagnostic performances of spectral CT parameters in AF, NP, and their differences in detecting degenerated disks.

		AUC values	Cutoff	Sensitivity (%)	Specificity (%)	Accuracy (%)
	N1	0.73 (0.68, 0.78)	≤97.9 HU	66.9 [123/184]	70.8 [109/154]	68.6 [232/338]
	N2	0.57 (0.52, 0.63)	>81.45 HU	61.4 [113/184]	53.3 [82/154]	57.7 [195/338]
	N3	0.62 (0.56, 0.67)	>80.35 HU	62.0 [114/184]	55.8 [86/154]	59.2 [200/338]
CT_conv_	N4	0.56 (0.51, 0.62)	>93.55 HU	23.9 [44/184]	91.6 [141/154]	54.7 [185/338]
	N5	0.68 (0.63, 0.73)	≤89.4 HU	53.3 [98/184]	80.5 [124/154]	65.7 [222/338]
	Δ(N1 − N3)	0.81 (0.76, 0.85)	≤14.75 HU	69.6 [128/184]	76.0 [117/154]	72.5 [245/338]
	Δ(N5 − N3)	0.77 (0.72, 0.81)	≤12.7 HU	76.1 [140/184]	66.9 [103/154]	71.9 [243/338]
	N1	0.76 (0.71, 0.80)	≤109.35%EDW	80.4 [148/184]	64.3 [99/154]	73.1 [232/338]
	N2	0.65 (0.59, 0.70)	>107.85%EDW	58.7 [108/184]	66.2 [102/154]	62.1 [210/338]
	N3	0.72 (0.67, 0.76)	>107.75%EDW	56.0 [103/184]	79.2 [122/154]	66.6 [225/338]
ED	N4	0.64 (0.59, 0.69)	>107. 50%EDW	62.5 [115/184]	59.7 [92/154]	61.2 [207/338]
	N5	0.71 (0.66, 0.76)	≤109.1%EDW	81.0 [149/184]	49.4 [76/154]	66.6 [225/338]
	Δ(N1 − N3)	0.92 (0.88, 0.94)	≤1.8%EDW	85.3 [157/184]	83.8 [129/154]	84.6 [286/338]
	Δ(N5 − N3)	0.88 (0.84, 0.91)	≤1.05%EDW	85.9 [158/184]	76.6 [118/154]	81.7 [276/338]
	N1	0.53 (0.48, 0.59)	≤7.35	70.7 [130/184]	38.3 [59/154]	55.9 [189/338]
	N2	0.66 (0.61, 0.71)	≤7.38	75.5 [139/184]	52.0 [80/154]	64.8 [219/338]
	N3	0.71 (0.66, 0.75)	≤7.36	64.1 [118/184]	71.4 [110/154]	67.5 [228/338]
Z_eff_	N4	0.65 (0.60, 0.70)	≤7.40	70.1 [129/184]	57.1 [88/154]	64.2 [217/338]
	N5	0.53 (0.47, 0.58)	≤7.27	21.7 [40/184]	89.6 [138/154]	52.7 [178/338]
	Δ(N1 − N3)	0.69 (0.64, 0.74)	>−0.03	52.7 [97/184]	77.9 [120/154]	64.2 [217/338]
	Δ(N5 − N3)	0.65 (0.60, 0.70)	>0.01	66.3 [122/184]	61.7 [95/154]	64.2 [217/338]

Data in parentheses are 95% confident intervals and those in brackets are numbers of patients. AF: annulus fibrosus; AUC: area under the receiver operating characteristic curve; CT_conv_: Conventional polyenergetic 120-kVp computed tomography value; ED: Electron density; %EDW: A relative value to the ED of water in percent; NP: Nucleus pulposus; Δ(N1 − N3): Difference between anterior AF and NP; Δ(N5 − N3): Difference between posterior AF and NP; Z_eff_: Effective atomic number.

**Fig 6:**
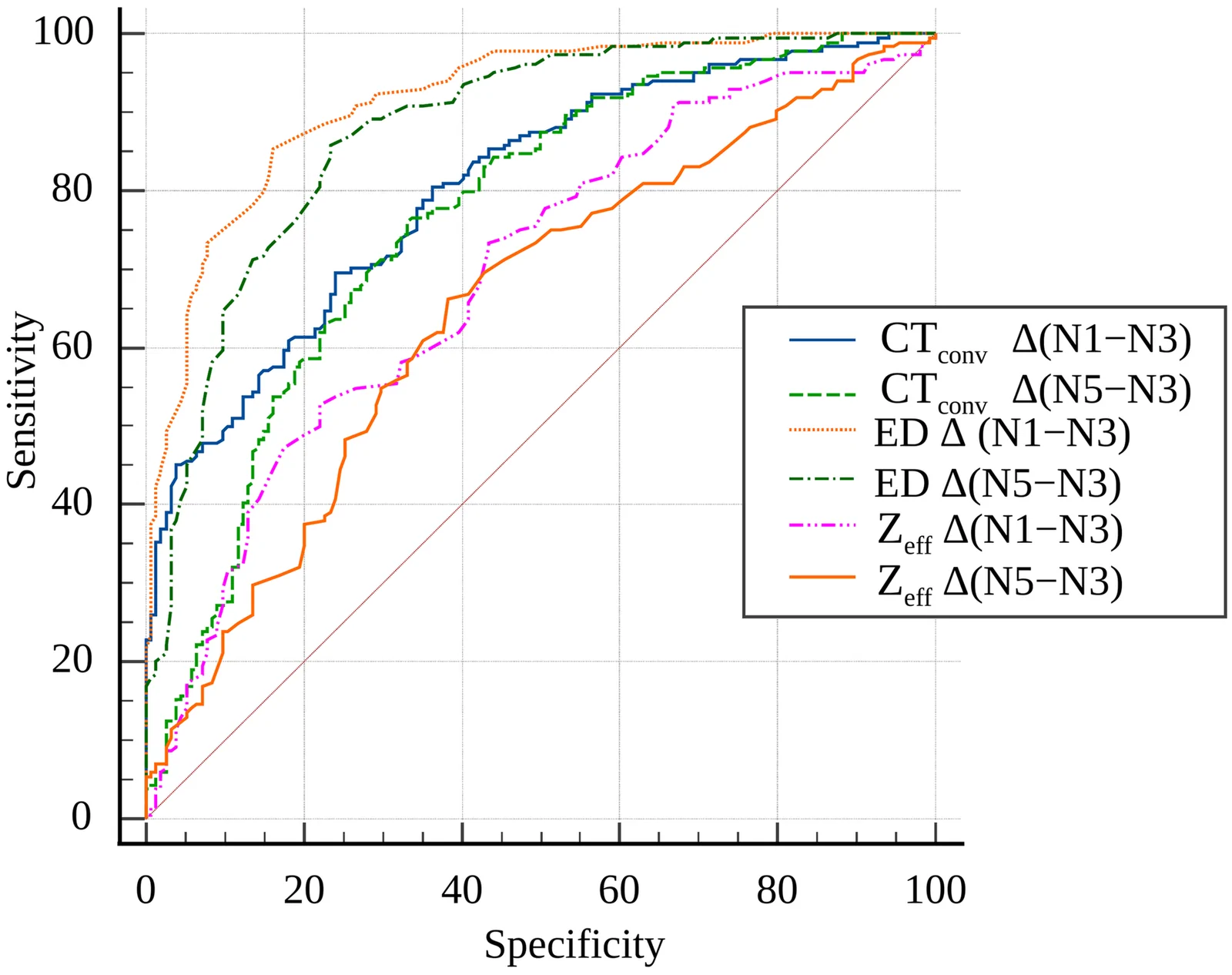
Receiver operating characteristic curves for differences of the CT_conv_, ED, and Z_eff_ on Δ(N1 − N3) and Δ(N5 − N3) for detecting degenerated disks.

The AUC values for Δ(N1 − N3) and Δ(N5 − N3) were 0.81 and 0.77 for CT_conv_, 0.92 and 0.88 for ED, and 0.69 and 0.65 for Z _eff_, respectively (Table 3). The DeLong method revealed significantly higher AUC values of ED for Δ(N1 − N3) and Δ(N5 − N3) (0.92 and 0.88) than those of CT_conv_ (0.81 and 0.77) and Z_eff_ (0.69 and 0.65) (*P* < 0.001–0.007) (Figs 6 and S3).

S3 Table summarizes the results of the other comparisons.

## Discussion

Low back pain is a symptom afflicting individuals globally, and one of the primary causes of low back pain is lumbar intervertebral disk degeneration. MRI is the predominant diagnostic tool for lumbar intervertebral disk degeneration, and the etiology of low back pain is multifaceted, making the identification of its cause difficult with only MRI. CT is useful for diagnosing other causes, but the use of CT for diagnosing lumbar intervertebral disk degeneration remains unknown. Our study revealed the potential usefulness of spectral CT parameters, particularly differences in ED value between the AF and the NP for evaluating lumbar intervertebral disk degeneration (AUC values of 0.92 and 0.88, each *P* < 0.001). These findings would contribute to the use of CT for assessing patients with low back pain, including those who cannot undergo MRI due to conditions, including long-term postural difficulty, cardiac pacemakers, or claustrophobia.

Mean ED values were positively weakly to moderately correlated with mPG in NP, whereas negatively weakly correlated with mPG in AF. ED differences between AF and NP demonstrated strong negative correlations with mPG and were significantly lower in degenerated disks than in normal disks. These indicate a sharp contrast on ED maps between AF and NP in normal disks, and this sharp contrast in normal disks diminishes in degenerated disks. The intervertebral disk consists of centrally located NP which consists of rich water contents, and peripherally located AF which is densely packed with fibrous components [4]. Intervertebral disk degeneration progression causes dehydration and acceleration of fibrosis in the NP, as well as cyst formation and tearing in the AF [5]. The ED value of water (100%EDW) is lower than that of fibrous tissue or connective tissue (approximately 110%EDW) [18,20,29]; thus, those pathologies would explain the increased ED in NP and decreased ED in AF in degenerated disks. Noteworthily, higher CT_conv_ and ED in AF were observed for mPG 5–8 compared to mPG 3 and 4 (Fig 4). It may indicate the presence of visually undetectable microscopic calcifications which generally increase with advancing grades of degeneration [30]. Previous studies have reported correlations between other spectral CT parameters, specifically VNC and collagen maps, and intervertebral disc degeneration. Shinohara et al. demonstrated that VNC values in NP showed a positive correlation with mPG (R^2^ = 0.574, *P* < 0.05), whereas no significant correlation was observed between VNC values and mPG in the annulus fibrosus (AF) (R^2^ = −0.015, *P* = 0.846) [10]. Pohlan et al reported that collagen map density in the anterior anulus fibrosus, but not in the nucleus pulposus, significantly declined with age (r = −0.09341, *p* = 0.0003), reflecting age-dependent reduction in collagen and proteoglycan content [15]. In this study, ED correlated with disc degeneration in both the NP and AF, and the difference in ED between these compartments showed a strong negative correlation with mPG. This contrasts with VNC, which correlated only with NP degeneration, and collagen maps, which reflected age-related changes only in the AF. Therefore, ED has the unique advantage of simultaneously assessing degenerative changes in both compartments of the intervertebral disc and may serve as a more comprehensive parameter for evaluating overall disc degeneration.

Our study revealed that ED was more closely correlated with mPG than CT_conv_ and Z_eff_ in almost all ROIs and the differences between AF and NP. Moreover, the AUC values of ED difference between AF and NP for disk degeneration detection were significantly higher than those of CT_conv_ and Z_eff_. These indicate that ED is a more useful disk degeneration indicator than CT_conv_ and Z_eff_. This may be due to the higher sensitivity of ED than the other two parameters to the above-mentioned pathological changes in degenerative disks. Additionally, noise suppression in ED images may partly explain the better detection of disk degeneration than the conventional polyenergetic CT images. Shinohara et al. reported that mean VNC values in the NP differed significantly between mPG grades (*P* < 0.05), except between grades 3 and 4 (*P* = 0.111), while mean VNC values in the AF showed no significant differences across mPG grades (*P* = 0.160–1.000) [10]. Regarding collagen maps, Bernatz et al. demonstrated an overall accuracy of 81% for visual assessment of disc degeneration [17]. While these spectral CT parameters, VNC and collagen maps, have proven useful in diagnosing disc degeneration, no studies to date, including the present investigation, have directly compared ED, VNC, and collagen maps in the context of disc degeneration. However, Jeong et al. directly compared ED, conventional CT, and VNC images for detecting lumbar disk herniation and reported that ED images achieved superior diagnostic performance due to lower coefficient of variation and higher normalized contrast ratio values [12]. This finding is consistent with our quantitative results demonstrating a stronger correlation between ED parameters and the degree of disk degeneration. Future studies should investigate whether comparative and integrated analyses of ED, VNC, and collagen mapping could further enhance diagnostic accuracy in the assessment of intervertebral disk degeneration

ED difference between anterior AF and NP demonstrated a stronger correlation with mPG and better performance in detecting disk degeneration than the ED difference between posterior AF and NP. These observations may be accounted for by the previously reported regional biochemical difference in AF that significantly decreased sulfated glycosaminoglycans concentration in anterior AF in an old-aged group with a higher Pfirrmann grade compared to the middle-aged group with a lower Pfirrmann grade, but no such difference was observed in the posterior AF [31].

The Z_eff_ value was negatively weakly correlated with mPG in NP but with no significant correlation with mPG in AF. The decrease in Z_eff_ in degenerated NP may be explained by loss of water, whose Z_eff_ value (approximately 7.68) is higher than that of fibrous tissue (approximately 7.30) [20,23,29]. Conversely, Z_eff_ was insensitive to AF degeneration. The lower performance of Z_eff_ in comparison with ED indicates the relatively limited usefulness of Z_eff_ in detecting disk degeneration.

Our study has some limitations. First, this was a single-center retrospective study involving patients with suspected spinal and spinal cord diseases, without healthy controls. The use of a single spectral CT platform (Philips Healthcare DLCT) may limit generalizability, and selection bias related to disease characteristics and severity cannot be excluded. Second, the gold standard for lumbar disk assessment in this study was mPG based on T2-weighted MRI, and we did not evaluate the histopathological change of disk degeneration. Third, inter-observer agreement for ROI measurements was moderate to substantial (mPG: weighted κ = 0.80; ED: ICC = 0.86; Z_eff_: ICC = 0.73; CT_conv_: ICC = 0.56), indicating suboptimal reproducibility. This may be attributed to manual ROI placement on single midsagittal images; volumetric measurements might improve reproducibility and accuracy. The lower agreement for CT_conv_ likely reflects inherent variability in conventional CT measurements rather than reader-related factors such as differences in radiological experience, as identical ROIs were used across all parameter maps. Fourth, the diagnostic performance of spectral CT did not reach the level of MRI, which remains the gold standard for disk degeneration assessment. Given the radiation exposure, our findings do not justify routine use of CT for disk degeneration assessment. At present, spectral CT may serve as an alternative imaging modality for patients with MRI contraindications (e.g., pacemakers, claustrophobia, metallic implants) or when simultaneous evaluation of other causes of low back pain is needed. Future multicenter prospective studies involving multiple spectral CT platforms from different vendors are warranted to validate inter-vendor reproducibility and establish the clinical utility of spectral CT parameters across diverse patient populations and technologies.

## Conclusions

we revealed the use of spectral CT parameters, especially ED, in assessing lumbar intervertebral disk degeneration. Our results may expand the role of CT in screening the cause of low back pain, potentially guiding more effective treatment strategies.

## Supporting information

S1 Table The sagittal T2-weighted imaging protocols. SENSE: Sensitivity encoding; GRAPPA: Generalized autocalibrating partially parallel acquisitions.
(DOCX)

S2 Table Complete crosstabulation for Diagnostic performances of Spectral CT parameters in AF, NP, and their differences in detecting degenerated disks. AF: Annulus fibrosus; AUC: Area under the receiver operating characteristic curve; CT_conv_: Conventional polyenergetic 120-kVp computed tomography value; DBE: Difference between areas; ED: Electron density; NP: Nucleus pulposus; Δ(N1 − N3): Difference between anterior AF and NP; Δ(N5 − N3): Difference between posterior AF and NP; Z_eff_: Effective atomic number.
(DOCX)

S3 Table Comparisons of AUC for Δ(N1 − N3) and Δ(N5 − N3) of spectral CT parameters for detecting degenerated disks. AF: Annulus fibrosus; AUC: Area under the receiver operating characteristic curve; CT_conv_: Conventional polyenergetic 120-kVp computed tomography value; DBE: Difference between areas; ED: Electron density; NP: Nucleus pulposus; Δ(N1 − N3): Difference between anterior AF and NP; Δ(N5 − N3): Difference between posterior AF and NP; Z_eff_: Effective atomic number.
(DOCX)

S4 Data Raw data underlying the results presented in this study. (XLS)

S5 Checklist The STARD reporting checklist. (DOCX)
