# Supplementary material for: Spectral CT electron density for evaluation of lumbar intervertebral disk degeneration
Source: PLoS One. 2026 Sep 1;21(9):e0357658. doi: 10.1371/journal.pone.0357658 (PMC13533366; doi:10.1371/journal.pone.0357658)
Supplement: Supplementary file 1 [file pone.0357658.s001.docx]

**S1 Table. The sagittal T2-weighted imaging protocols**

| Sequence Parameter | Achieva dStream | MAGNETOM Prisma |
| --- | --- | --- |
| Coil | Ds posterior coil | 32-channel spine coil |
| Pulse Sequence | Turbo Spin Echo | Turbo Spin Echo |
| Repetition time (msec) | 4000 | 4000 |
| Echo time (msec) | 76 | 80 |
| Flip angle (°) (Refocus°) | 90 (100) | 90 (120) |
| Field of view (mm) | 280 | 280 |
| Matrix (phase × frequency) | 260 × 400 | 314 × 448 |
| Turbo factor | 11 | 13 |
| Slice thickness (mm) | 3 | 3 |
| Slice number | 20 | 20 |
| Number of excitation | 1 | 1 |
| SENSE or GRAPPA | 1,8 | 2 |
| Scan time | 2 min 4 s | 1 min 48 s |

SENSE: Sensitivity encoding; GRAPPA: Generalized autocalibrating partially parallel acquisitions.
