# Supplementary material for: Spectral CT electron density for evaluation of lumbar intervertebral disk degeneration
Source: PLoS One. 2026 Sep 1;21(9):e0357658. doi: 10.1371/journal.pone.0357658 (PMC13533366; doi:10.1371/journal.pone.0357658)
Supplement: Supplementary file 2 [file pone.0357658.s002.docx]

**S2 Table. Complete crosstabulation for Diagnostic performances of DLCT parameters in AF, NP, and their differences in detecting degenerated disks.**

| CT_conv_ N1 | MRI Diagnosed as Degenerated disks | MRI Diagnosed as Normal disks | Total |
| --- | --- | --- | --- |
| Diagnosed as Degenerated disks | 123 | 45 | 168 |
| Diagnosed as Normal disks | 61 | 109 | 170 |
| Total | 184 | 154 | 338 |
| Sensitivity (%) | 66.9 | Specificity (%) | 70.8 |
| Positive Predictive Value (%) | 73.2 | Negative Predictive Value (%) | 64.1 |
|  |  | Accuracy (%) | 68.6 |

| CT_conv_ N2 | MRI Diagnosed as Degenerated disks | MRI Diagnosed as Normal disks | Total |
| --- | --- | --- | --- |
| Diagnosed as Degenerated disks | 113 | 72 | 185 |
| Diagnosed as Normal disks | 71 | 82 | 153 |
| Total | 184 | 154 | 338 |
| Sensitivity (%) | 61.4 | Specificity (%) | 53.3 |
| Positive Predictive Value (%) | 61.1 | Negative Predictive Value (%) | 53.6 |
|  |  | Accuracy (%) | 57.7 |

| CT_conv_ N3 | MRI Diagnosed as Degenerated disks | MRI Diagnosed as Normal disks | Total |
| --- | --- | --- | --- |
| Diagnosed as Degenerated disks | 114 | 68 | 182 |
| Diagnosed as Normal disks | 70 | 86 | 156 |
| Total | 184 | 154 | 338 |
| Sensitivity (%) | 62.0 | Specificity (%) | 55.8 |
| Positive Predictive Value (%) | 62.6 | Negative Predictive Value (%) | 55.1 |
|  |  | Accuracy (%) | 59.2 |

| CT_conv_ N4 | MRI Diagnosed as Degenerated disks | MRI Diagnosed as Normal disks | Total |
| --- | --- | --- | --- |
| Diagnosed as Degenerated disks | 44 | 13 | 57 |
| Diagnosed as Normal disks | 140 | 141 | 281 |
| Total | 184 | 154 | 338 |
| Sensitivity (%) | 23.9 | Specificity (%) | 91.6 |
| Positive Predictive Value (%) | 77.2 | Negative Predictive Value (%) | 50.2 |
|  |  | Accuracy (%) | 54.7 |

| CT_conv_ N5 | MRI Diagnosed as Degenerated disks | MRI Diagnosed as Normal disks | Total |
| --- | --- | --- | --- |
| Diagnosed as Degenerated disks | 98 | 30 | 128 |
| Diagnosed as Normal disks | 86 | 124 | 210 |
| Total | 184 | 154 | 338 |
| Sensitivity (%) | 53.3 | Specificity (%) | 80.5 |
| Positive Predictive Value (%) | 76.6 | Negative Predictive Value (%) | 59.0 |
|  |  | Accuracy (%) | 65.7 |

| CT_conv_ Δ(N1−N3) | MRI Diagnosed as Degenerated disks | MRI Diagnosed as Normal disks | Total |
| --- | --- | --- | --- |
| Diagnosed as Degenerated disks | 128 | 37 | 165 |
| Diagnosed as Normal disks | 56 | 117 | 173 |
| Total | 184 | 154 | 338 |
| Sensitivity (%) | 69.6 | Specificity (%) | 76.0 |
| Positive Predictive Value (%) | 77.6 | Negative Predictive Value (%) | 67.6 |
|  |  | Accuracy (%) | 72.5 |

| CT_conv_ Δ(N5−N3) | MRI Diagnosed as Degenerated disks | MRI Diagnosed as Normal disks | Total |
| --- | --- | --- | --- |
| Diagnosed as Degenerated disks | 140 | 51 | 191 |
| Diagnosed as Normal disks | 44 | 103 | 147 |
| Total | 184 | 154 | 338 |
| Sensitivity (%) | 76.1 | Specificity (%) | 66.9 |
| Positive Predictive Value (%) | 73.3 | Negative Predictive Value (%) | 70.1 |
|  |  | Accuracy (%) | 71.9 |

| ED N1 | MRI Diagnosed as Degenerated disks | MRI Diagnosed as Normal disks | Total |
| --- | --- | --- | --- |
| Diagnosed as Degenerated disks | 148 | 55 | 203 |
| Diagnosed as Normal disks | 36 | 99 | 135 |
| Total | 184 | 154 | 338 |
| Sensitivity (%) | 80.4 | Specificity (%) | 64.3 |
| Positive Predictive Value (%) | 72.9 | Negative Predictive Value (%) | 73.3 |
|  |  | Accuracy (%) | 73.1 |

| ED N2 | MRI Diagnosed as Degenerated disks | MRI Diagnosed as Normal disks | Total |
| --- | --- | --- | --- |
| Diagnosed as Degenerated disks | 108 | 52 | 160 |
| Diagnosed as Normal disks | 76 | 102 | 178 |
| Total | 184 | 154 | 338 |
| Sensitivity (%) | 58.7 | Specificity (%) | 66.2 |
| Positive Predictive Value (%) | 67.5 | Negative Predictive Value (%) | 57.3 |
|  |  | Accuracy (%) | 62.1 |

| ED N3 | MRI Diagnosed as Degenerated disks | MRI Diagnosed as Normal disks | Total |
| --- | --- | --- | --- |
| Diagnosed as Degenerated disks | 103 | 32 | 135 |
| Diagnosed as Normal disks | 81 | 122 | 203 |
| Total | 184 | 154 | 338 |
| Sensitivity (%) | 56.0 | Specificity (%) | 79.2 |
| Positive Predictive Value (%) | 76.3 | Negative Predictive Value (%) | 60.1 |
|  |  | Accuracy (%) | 66.6 |

| ED N4 | MRI Diagnosed as Degenerated disks | MRI Diagnosed as Normal disks | Total |
| --- | --- | --- | --- |
| Diagnosed as Degenerated disks | 115 | 62 | 177 |
| Diagnosed as Normal disks | 69 | 92 | 161 |
| Total | 184 | 154 | 338 |
| Sensitivity (%) | 62.5 | Specificity (%) | 59.7 |
| Positive Predictive Value (%) | 65.0 | Negative Predictive Value (%) | 57.1 |
|  |  | Accuracy (%) | 61.2 |

| ED N5 | MRI Diagnosed as Degenerated disks | MRI Diagnosed as Normal disks | Total |
| --- | --- | --- | --- |
| Diagnosed as Degenerated disks | 149 | 78 | 227 |
| Diagnosed as Normal disks | 35 | 76 | 111 |
| Total | 184 | 154 | 338 |
| Sensitivity (%) | 81.0 | Specificity (%) | 49.4 |
| Positive Predictive Value (%) | 65.6 | Negative Predictive Value (%) | 68.5 |
|  |  | Accuracy (%) | 66.6 |

| ED Δ(N1−N3) | MRI Diagnosed as Degenerated disks | MRI Diagnosed as Normal disks | Total |
| --- | --- | --- | --- |
| Diagnosed as Degenerated disks | 157 | 25 | 182 |
| Diagnosed as Normal disks | 27 | 129 | 156 |
| Total | 184 | 154 | 338 |
| Sensitivity (%) | 85.3 | Specificity (%) | 83.8 |
| Positive Predictive Value (%) | 86.3 | Negative Predictive Value (%) | 82.7 |
|  |  | Accuracy (%) | 84.6 |

| ED Δ(N5−N3) | MRI Diagnosed as Degenerated disks | MRI Diagnosed as Normal disks | Total |
| --- | --- | --- | --- |
| Diagnosed as Degenerated disks | 158 | 36 | 194 |
| Diagnosed as Normal disks | 26 | 118 | 144 |
| Total | 184 | 154 | 338 |
| Sensitivity (%) | 85.9 | Specificity (%) | 76.6 |
| Positive Predictive Value (%) | 81.4 | Negative Predictive Value (%) | 81.9 |
|  |  | Accuracy (%) | 81.7 |

| Z_eff_ N1 | MRI Diagnosed as Degenerated disks | MRI Diagnosed as Normal disks | Total |
| --- | --- | --- | --- |
| Diagnosed as Degenerated disks | 130 | 95 | 225 |
| Diagnosed as Normal disks | 54 | 59 | 113 |
| Total | 184 | 154 | 338 |
| Sensitivity (%) | 70.7 | Specificity (%) | 38.3 |
| Positive Predictive Value (%) | 57.8 | Negative Predictive Value (%) | 52.2 |
|  |  | Accuracy (%) | 55.9 |

| Z_eff_ N2 | MRI Diagnosed as Degenerated disks | MRI Diagnosed as Normal disks | Total |
| --- | --- | --- | --- |
| Diagnosed as Degenerated disks | 139 | 74 | 213 |
| Diagnosed as Normal disks | 45 | 80 | 125 |
| Total | 184 | 154 | 338 |
| Sensitivity (%) | 75.5 | Specificity (%) | 51.9 |
| Positive Predictive Value (%) | 65.3 | Negative Predictive Value (%) | 64.0 |
|  |  | Accuracy (%) | 64.8 |

| Z_eff_ N3 | MRI Diagnosed as Degenerated disks | MRI Diagnosed as Normal disks | Total |
| --- | --- | --- | --- |
| Diagnosed as Degenerated disks | 118 | 44 | 162 |
| Diagnosed as Normal disks | 66 | 110 | 176 |
| Total | 184 | 154 | 338 |
| Sensitivity (%) | 64.1 | Specificity (%) | 71.4 |
| Positive Predictive Value (%) | 72.8 | Negative Predictive Value (%) | 62.5 |
|  |  | Accuracy (%) | 67.5 |

| Z_eff_ N4 | MRI Diagnosed as Degenerated disks | MRI Diagnosed as Normal disks | Total |
| --- | --- | --- | --- |
| Diagnosed as Degenerated disks | 129 | 66 | 195 |
| Diagnosed as Normal disks | 55 | 88 | 143 |
| Total | 184 | 154 | 338 |
| Sensitivity (%) | 70.1 | Specificity (%) | 57.1 |
| Positive Predictive Value (%) | 66.2 | Negative Predictive Value (%) | 61.5 |
|  |  | Accuracy (%) | 64.2 |

| Z_eff_ N5 | MRI Diagnosed as Degenerated disks | MRI Diagnosed as Normal disks | Total |
| --- | --- | --- | --- |
| Diagnosed as Degenerated disks | 40 | 16 | 56 |
| Diagnosed as Normal disks | 144 | 138 | 282 |
| Total | 184 | 154 | 338 |
| Sensitivity (%) | 21.7 | Specificity (%) | 89.6 |
| Positive Predictive Value (%) | 71.4 | Negative Predictive Value (%) | 48.9 |
|  |  | Accuracy (%) | 52.7 |

| Z_eff_ Δ(N1−N3) | MRI Diagnosed as Degenerated disks | MRI Diagnosed as Normal disks | Total |
| --- | --- | --- | --- |
| Diagnosed as Degenerated disks | 97 | 34 | 131 |
| Diagnosed as Normal disks | 87 | 120 | 207 |
| Total | 184 | 154 | 338 |
| Sensitivity (%) | 52.7 | Specificity (%) | 77.9 |
| Positive Predictive Value (%) | 74.0 | Negative Predictive Value (%) | 58.0 |
|  |  | Accuracy (%) | 64.2 |

| Z_eff_ Δ(N1−N3) | MRI Diagnosed as Degenerated disks | MRI Diagnosed as Normal disks | Total |
| --- | --- | --- | --- |
| Diagnosed as Degenerated disks | 122 | 59 | 181 |
| Diagnosed as Normal disks | 62 | 95 | 157 |
| Total | 184 | 154 | 338 |
| Sensitivity (%) | 66.3 | Specificity (%) | 61.7 |
| Positive Predictive Value (%) | 67.4 | Negative Predictive Value (%) | 60.5 |
|  |  | Accuracy (%) | 64.2 |
