# Supplementary material for: Spectral CT electron density for evaluation of lumbar intervertebral disk degeneration
Source: PLoS One. 2026 Sep 1;21(9):e0357658. doi: 10.1371/journal.pone.0357658 (PMC13533366; doi:10.1371/journal.pone.0357658)
Supplement: Supplementary file 3 [file pone.0357658.s003.docx]

**S3 Table. Comparisons of AUC for *Δ(N1−N3)* and *Δ(N5−N3)* of DECT parameters for detecting degenerated disks**

|  | CT_conv_ Δ(N5−N3) | ED Δ(N1−N3) | ED Δ(N5−N3) | Z_eff_ Δ(N1−N3) | Z_eff_ Δ(N5−N3) |
| --- | --- | --- | --- | --- | --- |
| CT_conv_ Δ(N1−N3) |  |  |  |  |  |
| DBE | 0.04 | 0.11 | 0.07 | 0.12 | 0.16 |
| *P*-value | 0.122 | <0.001 | 0.007 | 0.005 | <0.001 |
| CT_conv_ Δ(N5−N3) |  |  |  |  |  |
| DBE |  | 0.15 | 0.11 | 0.08 | 0.12 |
| *P*-value |  | <0.001 | <0.001 | 0.059 | 0.008 |
| ED Δ(N1−N3) |  |  |  |  |  |
| DBE |  |  | 0.04 | 0.23 | 0.27 |
| *P*-value |  |  | 0.06 | <0.001 | <0.001 |
| ED Δ(N5−N3) |  |  |  |  |  |
| DBE |  |  |  | 0.19 | 0.23 |
| *P*-value |  |  |  | <0.001 | <0.001 |
| Z_eff_ Δ(N1−N3) |  |  |  |  |  |
| DBE |  |  |  |  | 0.04 |
| *P*-value |  |  |  |  | 0.258 |

AF: Annulus fibrosus; AUC: Area under the receiver operating characteristic curve;  CT_conv_: Conventional polyenergetic 120-kVp computed tomography value; DBE: Difference between areas; DECT: Dual-energy computed tomography; ED: Electron density; NP: Nucleus pulposus; Δ(N1−N3): Difference between anterior AF and NP; Δ(N5−N3): Difference between posterior AF and NP; Z_eff_: Effective atomic number.
