# Supplementary material for: Spectral CT electron density for evaluation of lumbar intervertebral disk degeneration
Source: PLoS One. 2026 Sep 1;21(9):e0357658. doi: 10.1371/journal.pone.0357658 (PMC13533366; doi:10.1371/journal.pone.0357658)
Supplement: Supplementary file 4 [file pone.0357658.s005.docx]

The STARD reporting checklist

For checking that diagnostic accuracy study articles can be understood and used by everyone

 Note

If you have not used a reporting guideline before, read about [how and why to use them](https:/resources.equator-network.org/about/reporting-guidelines.html) and check whether STARD is the [most applicable reporting guideline](https:/resources.equator-network.org/reporting-guidelines/stard/index.html?#applicability) for your work.

Reporting guidelines are most useful when used early in research. When writing a manuscript or application, consider using the [Full Guidance](https:/resources.equator-network.org/reporting-guidelines/stard/index.html) where you’ll see explanations and examples for each item.

After writing, demonstrate adherence by completing this checklist:

1. Specify where each item is described (see Note 1).
2. Cite this checklist (See Note 2).
3. Include your completed checklist as a supplement when submitting to a journal so that future readers can use it to find information.

|  | Item Description | Location (or reason for not reporting) |
| --- | --- | --- |
| **Title or abstract** |  |  |
| [1. Identification as a study of diagnostic accuracy](https:/resources.equator-network.org/reporting-guidelines/stard/items/identification.html?utm_source=stard&utm_medium=checklist&utm_campaign=STARD_2015_v1_1) | Identification as a study of diagnostic accuracy using at least one measure of accuracy (such as sensitivity, specificity, predictive values, or AUC). | Result, Fig 5, S2, and Table 3. |
| **Abstract** |  |  |
| [2. Abstract](https:/resources.equator-network.org/reporting-guidelines/stard/items/abstract.html?utm_source=stard&utm_medium=checklist&utm_campaign=STARD_2015_v1_1) | Structured summary of study design, methods, results and conclusions (for specific guidance, see STARD for Abstracts). | Abstract: Objective, Summary of Background Data, Methods, Results, and Conclusion sections.(Page2, line3-Page3, line2) |
| **Introduction** |  |  |
| [3. Background](https:/resources.equator-network.org/reporting-guidelines/stard/items/background.html?utm_source=stard&utm_medium=checklist&utm_campaign=STARD_2015_v1_1) | Scientific and clinical background, including the intended use and clinical role of the index test. | Introduction section, Page 3 line 4 - page 4, line 10 |
| [4. Objectives](https:/resources.equator-network.org/reporting-guidelines/stard/items/objectives.html?utm_source=stard&utm_medium=checklist&utm_campaign=STARD_2015_v1_1) | Study objectives and hypotheses. | Introduction section, Page 4, line 10-12 |
| **Methods** |  |  |
| [5. Study design](https:/resources.equator-network.org/reporting-guidelines/stard/items/study-design.html?utm_source=stard&utm_medium=checklist&utm_campaign=STARD_2015_v1_1) | Whether data collection was planned before the index test and reference standard were performed (prospective study) or after (retrospective study). | Material and Methods, Patient population, Page 4, line 15- Page 5, line 1 |
| Participants |  |  |
| [6. Eligibility criteria](https:/resources.equator-network.org/reporting-guidelines/stard/items/eligibility-criteria.html?utm_source=stard&utm_medium=checklist&utm_campaign=STARD_2015_v1_1) | Eligibility criteria. | Material and Methods, Patient population, page 5, line 4-9 |
| [7. Identifying eligible participants](https:/resources.equator-network.org/reporting-guidelines/stard/items/identifying-eligible-participants.html?utm_source=stard&utm_medium=checklist&utm_campaign=STARD_2015_v1_1) | On what basis potentially eligible participants were identified (such as symptoms, results from previous tests, inclusion in registry). | Material and Methods, Patient population, page 5, line 4-9 |
| [8. Setting, location, and dates](https:/resources.equator-network.org/reporting-guidelines/stard/items/setting-location-dates.html?utm_source=stard&utm_medium=checklist&utm_campaign=STARD_2015_v1_1) | Where and when potentially eligible participants were identified (setting, location and dates). | Material and Methods, Patient population, page 5, line 1 |
| [9. Consecutive, random or convenience series](https:/resources.equator-network.org/reporting-guidelines/stard/items/consecutive-random-convenience-series.html?utm_source=stard&utm_medium=checklist&utm_campaign=STARD_2015_v1_1) | Whether participants formed a consecutive, random or convenience series. | Material and Methods, Patient population, page 5, line 3 |
| Test Methods |  |  |
| [10. Index test & Reference standard](https:/resources.equator-network.org/reporting-guidelines/stard/items/index-test.html?utm_source=stard&utm_medium=checklist&utm_campaign=STARD_2015_v1_1) | 10a. Index test  10b. Reference standard | 10a: Material and Methods, Spectral CT imaging protocols, page 6, line 1-3  10b: Material and Methods, Image analysis, page 6, line 12-14 |
| [11. Reference standard rationale](https:/resources.equator-network.org/reporting-guidelines/stard/items/reference-standard-rationale.html?utm_source=stard&utm_medium=checklist&utm_campaign=STARD_2015_v1_1) | Rationale for choosing the reference standard (if alternatives exist). | References 6 and 7, page 23, line 14- page 24, line 2 |
| [12. Index test and reference standard cut-offs or categories](https:/resources.equator-network.org/reporting-guidelines/stard/items/index-test-cut-offs-categories.html?utm_source=stard&utm_medium=checklist&utm_campaign=STARD_2015_v1_1) | 12a. Definition of and rationale for test positivity cut-offs or result categories of the index test, distinguishing prespecified from exploratory.  12b. Definition of and rationale for test positivity cut-offs or result categories of the reference standard, distinguishing prespecified from exploratory. | Material and Methods, Image analysis, Statistical analysis, page 6 line 9 - page 8 line 6 |
| [13. Information available to performers or readers of the index test and reference standard assessors](https:/resources.equator-network.org/reporting-guidelines/stard/items/information-available-index.html?utm_source=stard&utm_medium=checklist&utm_campaign=STARD_2015_v1_1) | 13a. Whether clinical information and reference standard results were available to the performers or readers of the index test.  13b. Whether clinical information and index test results were available to the assessors of the reference standard. | Material and Methods, Image analysis, page 6, line 10-11 |
| Analysis |  |  |
| [14. Analysis methods](https:/resources.equator-network.org/reporting-guidelines/stard/items/analysis-methods.html?utm_source=stard&utm_medium=checklist&utm_campaign=STARD_2015_v1_1) | Methods for estimating or comparing measures of diagnostic accuracy. | Material and Methods, Statistical analysis, page 7 line 6 - page 8 line 6 |
| [15. Indeterminate results](https:/resources.equator-network.org/reporting-guidelines/stard/items/indeterminate-results.html?utm_source=stard&utm_medium=checklist&utm_campaign=STARD_2015_v1_1) | How indeterminate index test or reference standard results were handled. | Material and Methods, Patient population, page 5, line 6-10 |
| [16. Missing data](https:/resources.equator-network.org/reporting-guidelines/stard/items/missing-data.html?utm_source=stard&utm_medium=checklist&utm_campaign=STARD_2015_v1_1) | How missing data on the index test and reference standard were handled. | Material and Methods, Patient population, page 5, line 2-7 |
| [17. Variability](https:/resources.equator-network.org/reporting-guidelines/stard/items/variability.html?utm_source=stard&utm_medium=checklist&utm_campaign=STARD_2015_v1_1) | Any analyses of variability in diagnostic accuracy, distinguishing prespecified from exploratory. | Not Applicable |
| [18. Intended sample size](https:/resources.equator-network.org/reporting-guidelines/stard/items/intended-sample-size.html?utm_source=stard&utm_medium=checklist&utm_campaign=STARD_2015_v1_1) | Intended sample size and how it was determined. | Not Applicable |
| **Results** |  |  |
| Participants |  |  |
| [19. Participant flow diagram](https:/resources.equator-network.org/reporting-guidelines/stard/items/flow-diagram.html?utm_source=stard&utm_medium=checklist&utm_campaign=STARD_2015_v1_1) | Flow of participants, using a diagram. | Material and Methods, Patient population, Fig 1 |
| [20. Baseline characteristics](https:/resources.equator-network.org/reporting-guidelines/stard/items/baseline-characteristics.html?utm_source=stard&utm_medium=checklist&utm_campaign=STARD_2015_v1_1) | Baseline demographic and clinical characteristics of participants. | Results, Patient characteristics, page 8, line 9-10. Table 1. |
| [21a. Participants with and without the target condition](https:/resources.equator-network.org/reporting-guidelines/stard/items/participants-with-target-condition.html?utm_source=stard&utm_medium=checklist&utm_campaign=STARD_2015_v1_1) | 21a. Distribution of severity of disease in those with the target condition.  21b. Distribution of alternative diagnoses in those without the target condition | Results, The mPG of lumbar intervertebral disks, page 11, line 9-11. |
| [22. Time interval](https:/resources.equator-network.org/reporting-guidelines/stard/items/time-interval.html?utm_source=stard&utm_medium=checklist&utm_campaign=STARD_2015_v1_1) | Time interval and any clinical interventions between index test and reference standard. | Material and Methods, Patient population, page 5, line 4 |
| Test Results |  |  |
| [23. Index test and reference standard results](https:/resources.equator-network.org/reporting-guidelines/stard/items/index-reference-results.html?utm_source=stard&utm_medium=checklist&utm_campaign=STARD_2015_v1_1) | Cross tabulation of the index test results (or their distribution) by the results of the reference standard. | Results, Comparison of spectral CT parameters between normal and degenerated disks, S2 Table. |
| [24. Estimates of accuracy](https:/resources.equator-network.org/reporting-guidelines/stard/items/estimates-of-accuracy.html?utm_source=stard&utm_medium=checklist&utm_campaign=STARD_2015_v1_1) | Estimates of diagnostic accuracy and their precision (such as 95% CIs). | Results, Inter-observer agreements of mPG and spectral CT parameters, page 12, line 2-4  Results, Correlation between spectral CT parameters and lumbar intervertebral disk degeneration, page 14, line 7-page 15, line 3.  Results, Comparison of spectral CT parameters between normal and degenerated disks, page 15, line 4-page 16, line 3.  Results, Diagnostic performances of spectral CT parameters in differentiating degenerated disks from normal disks, Table3. page 16, line 4-page 18, line 7. |
| [25. Adverse events](https:/resources.equator-network.org/reporting-guidelines/stard/items/adverse-events.html?utm_source=stard&utm_medium=checklist&utm_campaign=STARD_2015_v1_1) | Any adverse events from performing the index test or the reference standard. | Not Applicable |
| **Discussion** |  |  |
| [26. Limitations](https:/resources.equator-network.org/reporting-guidelines/stard/items/limitations.html?utm_source=stard&utm_medium=checklist&utm_campaign=STARD_2015_v1_1) | Study limitations, including sources of potential bias, statistical uncertainty and generalisability. | Discussion, page 21 line 13- page 22 line 10 |
| [27. Implications for Practice](https:/resources.equator-network.org/reporting-guidelines/stard/items/implications-for-practice.html?utm_source=stard&utm_medium=checklist&utm_campaign=STARD_2015_v1_1) | Implications for practice, including the intended use and clinical role of the index test. | Discussion, page 22, line 4-10 |
| **Other information** |  |  |
| [28. Registration](https:/resources.equator-network.org/reporting-guidelines/stard/items/registration.html?utm_source=stard&utm_medium=checklist&utm_campaign=STARD_2015_v1_1) | Registration number and name of registry. | Material and Methods, Patient population, page 4, line 15. |
| [29. Protocol](https:/resources.equator-network.org/reporting-guidelines/stard/items/protocol.html?utm_source=stard&utm_medium=checklist&utm_campaign=STARD_2015_v1_1) | Where the full study protocol can be accessed. | Ethics Committee on Clinical Research, Sakuragaoka Campus, Kagoshima University |
| [30. Funding](https:/resources.equator-network.org/reporting-guidelines/stard/items/funding.html?utm_source=stard&utm_medium=checklist&utm_campaign=STARD_2015_v1_1) | Sources of funding and other support; role of funders. | Financial Disclosure Statement, page 1, line 3-4 |

## 1 How to specify where content is

Tell the reader where they can find information. E.g.,

- Results; paragraph 2
- Methods, Participants; paragraphs 1 & 2.
- Table 3
- Supplement B, para. 4

If you have chosen not to describe an item, explain why. You can do this in the checklist, or as a note below it.

You can describe items in the article body, or in tables, figures, or supplementary materials, and should prioritize items you feel are most important to your intended audience. The order of items in your manuscript does not need to match the order of items in this checklist. You can decide how best to structure your work.

## 2 How to cite

Describe how you used STARD at the end of your Methods section, referencing the resources you used e.g.,

‘We used the STARD reporting guideline(1) to draft this manuscript, and the STARD reporting checklist(2) when editing, included in supplement A’

If you use a reporting checklist, remember to include it as a supplement when publishing so that readers can easily find information and see how you have interpreted the guidance.

1.	Bossuyt PM, Reitsma JB, Bruns DE, Gatsonis CA, Glasziou PP, Irwig L, et al. STARD 2015: An updated list of essential items for reporting diagnostic accuracy studies. BMJ [Internet]. 2015 Oct;351:h5527. Available from: <https://www.bmj.com/content/351/bmj.h5527>

2.	Bossuyt PM, Reitsma JB, Bruns DE, Gatsonis CA, Glasziou PP, Irwig L, et al. The STARD reporting checklist. In: Harwood J, Albury C, Beyer J de, Schlüssel M, Collins G, editors. The EQUATOR network reporting guideline platform [Internet]. The UK EQUATOR Centre; 2025. Available from: [https:/resources.equator-network.org/reporting-guidelines/stard/stard-checklist.docx](https://https:/resources.equator-network.org/reporting-guidelines/stard/stard-checklist.docx)
