# Supplementary material for: Spectral CT electron density for evaluation of lumbar intervertebral disk degeneration
Source: PLoS One. 2026 Sep 1;21(9):e0357658. doi: 10.1371/journal.pone.0357658 (PMC13533366; doi:10.1371/journal.pone.0357658)
Supplement: Supplementary file 5 [file pone.0357658.s007.doc]

**EDITOR’S COMMENTS:**

**[E# 1] The introduction section needs to be more focused to the research problem and the research question should be clearly implied within your introduction.**

We thank the editor for this comment and have deleted some sentences to focus on spectral CT and research problem.

New sentence: Lumbar intervertebral disk degeneration is the most prevalent cause of low back pain [1, 2], and it causes herniated disk disease, lumbar degenerative spondylosis, and lumbar spinal canal stenosis [3].

The intervertebral disk is centrally and peripherally composed of the nucleus pulposus (NP) and the annulus fibrosus (AF), respectively. The AF consists of a fibrocartilaginous structure that primarily contains type I collagen, whereas the NP is rich in water and proteoglycans in a loose network of type II collagen [4]. Proteoglycans in disk degeneration, which function to bind water, decrease, thereby causing dehydration and relatively increased type I collagen within the NP, and tears and degenerate cysts of AF [5]. Lumbar intervertebral disk degeneration evaluation is conventionally performed using Magnetic resonance imaging (MRI) [1, 2, 5–7]. Pfirrmann grade and modified Pfirrmann grade (mPG) based on T2-weighted images are predominantly used for grading degenerative disk disease [6, 7]. Thus, MRI is useful for intervertebral disk degeneration evaluation. However, MRI is unavailable for patients with contraindicated metal implantation or claustrophobia. Moreover, MRI may not be an optimal tool for diagnosing other causative diseases of lower back pain than spinal diseases such as urinary tract stones, aortic dissection, etc. Computed tomography (CT) effectively evaluated these diseases, but its usefulness for lumbar intervertebral disk degeneration evaluation has not been established.

Spectral CT imaging encompasses a unique generation of CT systems that utilize energy-dependent information, with dual-energy CT (DECT) representing its first generation, which can be implemented through various technical approaches including dual-source CT, rapid kVp-switching, and dual-layer spectral detector CT (DLCT) [8]. DECT analyzes data of Compton scattering and the photoelectric effect of X–rays of two energies using various algorithms. DECT studies of intervertebral disk diseases have been reported in recent years, specifically including virtual non-calcium imaging for disk herniation and degeneration [9–13], water imaging for disk degeneration [14], collagen mapping for disk degeneration [15–17], and electron density (ED) imaging for disk herniation [12, 13]. ED and effective atomic number (Z_eff_) are recently available as DECT derived parameters [18]. DECT enable more accurate measurements of ED and Z_eff_ through improved dose distribution accuracy, which were difficult to obtain with conventional single-energy CT [19]. While these measurements have been primarily investigated for improving dose calculation accuracy in radiation treatment planning [20], their utility in diagnostic imaging has remained underexplored. Recently, however, the diagnostic value of ED for evaluating disk herniation has been reported [12, 13]. To the best of our knowledge, the use of ED and Z_eff_ images for lumbar intervertebral disk degeneration evaluation remains unknown. Therefore, this study aimed to investigate whether spectral CT-derived ED and Zeff can accurately differentiate varying degrees of lumbar disk degeneration. (P3, line4- P4, line12).

**[E# 2]Second, you need to discuss the limitations around generalizability of your findings.**

We thank the editor for this comment and added the following sentences as a limitation in Discussion according to Editor’s suggestion.

New sentence: First, this was a single-center retrospective study involving patients with suspected spinal and spinal cord diseases, without healthy controls. The use of a single spectral CT platform (Philips Healthcare DLCT) may limit generalizability, and selection bias related to disease characteristics and severity cannot be excluded. (P21,line13-15).

**REVIEWER’S COMMENTS**

**Reviewer# 1**

**I have carefully reviewed the manuscript and find it to be well written and scientifically sound. The objective is clearly stated, achievable, and the findings contribute meaningfully to the growing body of knowledge in computed tomography (CT) science. The study also highlights novel potential applications of CT technology. However, a few issues require clarification or further elaboration:**

**[R# 1-1] 1. CT Dose Justification:**

**Did the researcher consider patient radiation dose during CT examinations for intervertebral degeneration assessment? Please clarify whether the use of CT for this indication is justified in terms of benefit versus exposure.**

We thank the reviewer for this comment and added the following sentences as a limitation in Discussion according to reviewer’s suggestion.

New sentence: Fourth, the diagnostic performance of spectral CT did not reach the level of MRI, which remains the gold standard for disk degeneration assessment. Given the radiation exposure, our findings do not justify routine use of CT for disk degeneration assessment. At present, spectral CT may serve as an alternative imaging modality for patients with MRI contraindications (e.g., pacemakers, claustrophobia, metallic implants) or when simultaneous evaluation of other causes of low back pain is needed. Future multicenter prospective studies involving multiple spectral CT platforms from different vendors are warranted to validate inter-vendor reproducibility and establish the clinical utility of spectral CT parameters across diverse patient populations and technologies. (P22, line4-10).

**[R# 1-2] 2. Introduction Contextual Clarity:**

**The introduction mentions that DECT studies of intervertebral disk diseases have been reported in recent years. Although this statement is referenced, it would strengthen the background if specific examples of such disk diseases were mentioned. Additionally, the author should explain why effective atomic number (Z_eff_) and electron density (ED) images for lumbar intervertebral disk degeneration (LIDD) remain underexplored. Are there particular research or technical limitations contributing to this gap?**

We thank the reviewer for this comment and added the following sentences in Introduction according to reviewer’s suggestion.

New sentence: DECT studies of intervertebral disk diseases have been reported in recent years, specifically including virtual non-calcium imaging for disk herniation and degeneration [9–13], water imaging for disk degeneration [14], collagen mapping for disk degeneration [15–17], and electron density (ED) imaging for disk herniation [12, 13]. ED and effective atomic number (Z_eff_) are recently available as DECT derived parameters [18]. DECT enable more accurate measurements of ED and Z_eff_ through improved dose distribution accuracy, which were difficult to obtain with conventional single-energy CT [19]. While these measurements have been primarily investigated for improving dose calculation accuracy in radiation treatment planning [20], their utility in diagnostic imaging has remained underexplored. Recently, however, the diagnostic value of ED for evaluating disk herniation has been reported [12, 13] (P4, line4-10).

**[R# 1-3]3. Methods Reader Experience:**

**Under the analysis section, there appears to be a considerable difference in professional experience between the two radiologists (8 years vs. 17 years). Please clarify whether both radiologists possess similar expertise in DECT interpretation and whether this variation could have influenced the image reading accuracy or overall results.**

We thank the reviewer for this comment and added the following sentences as a limitation in Discussion according to reviewer’s suggestion.

New sentence: Third, inter-observer agreement for ROI measurements was moderate to substantial (mPG: weighted κ = 0.80; ED: ICC = 0.86; Z_eff_: ICC = 0.73; CT_conv_: ICC = 0.56), indicating suboptimal reproducibility. This may be attributed to manual ROI placement on single midsagittal images; volumetric measurements might improve reproducibility and accuracy. The lower agreement for CT_conv_ likely reflects inherent variability in conventional CT measurements rather than reader-related factors such as differences in radiological experience, as identical ROIs were used across all parameter maps. (P21, line16-P22, line4).

**[R# 1-4]4. Generalisability:**

**The study appears to have been conducted at a single center. The authors should discuss how this limitation might affect the generalisability of the findings to other clinical settings or populations.**

We thank the reviewer for this comment and added the following sentences as a limitation in Discussion according to reviewer’s suggestion.

New sentence: First, this was a single-center retrospective study involving patients with suspected spinal and spinal cord diseases, without healthy controls. The use of a single spectral CT platform (Philips Healthcare DLCT) may limit generalizability, and selection bias related to disease characteristics and severity cannot be excluded. (P21,line13-15).

**[R# 1-5]5. Clinical Applicability:**

**Based on the study outcomes, do the authors recommend adopting this DECT-based method for clinical assessment of intervertebral disk degeneration, or is further validation and research still warranted before routine clinical implementation?**

**Overall, this is a well-conceived and informative paper, and with the above clarifications, it could make a valuable contribution to the field.**

We thank the reviewer for this comment and added the following sentences as a limitation in Discussion according to reviewer’s suggestion.

New sentence: Fourth, the diagnostic performance of spectral CT did not reach the level of MRI, which remains the gold standard for disk degeneration assessment. Given the radiation exposure, our findings do not justify routine use of CT for disk degeneration assessment. At present, spectral CT may serve as an alternative imaging modality for patients with MRI contraindications (e.g., pacemakers, claustrophobia, metallic implants) or when simultaneous evaluation of other causes of low back pain is needed. Future multicenter prospective studies involving multiple spectral CT platforms from different vendors are warranted to validate inter-vendor reproducibility and establish the clinical utility of spectral CT parameters across diverse patient populations and technologies. (P21,line4-10).

**Reviewer #2:**

**The authors present a study on spectral CT for assessing lumbar disc degeneration, utilizing basic parameters such as electron density (ED) and effective atomic number (Zeff) to characterize the intervertebral disc. However, they overlook a comparison or discussion of previously reported techniques, such as VNCa and collagen mapping. The results and discussion sections are lengthy and lack organization. Additionally, the authors miss the opportunity to clearly articulate the hypothesis of their work in the introduction. Below are further specific points for consideration.**

****Major Points:****

**[R#2-1] The introduction is overly broad and lacks specificity, reiterating established facts. I recommend streamlining it to focus on spectral CT and discussing why the authors believe electron density is a valuable target, especially since this approach has not been previously explored in depth**.

We thank the reviewer for this comment and have deleted some sentences to focus on spectral CT and research problem.

New sentence: Lumbar intervertebral disk degeneration is the most prevalent cause of low back pain [1, 2], and it causes herniated disk disease, lumbar degenerative spondylosis, and lumbar spinal canal stenosis [3].

The intervertebral disk is centrally and peripherally composed of the nucleus pulposus (NP) and the annulus fibrosus (AF), respectively. The AF consists of a fibrocartilaginous structure that primarily contains type I collagen, whereas the NP is rich in water and proteoglycans in a loose network of type II collagen [4]. Proteoglycans in disk degeneration, which function to bind water, decrease, thereby causing dehydration and relatively increased type I collagen within the NP, and tears and degenerate cysts of AF [5]. Lumbar intervertebral disk degeneration evaluation is conventionally performed using Magnetic resonance imaging (MRI) [1, 2, 5–7]. Pfirrmann grade and modified Pfirrmann grade (mPG) based on T2-weighted images are predominantly used for grading degenerative disk disease [6, 7]. Thus, MRI is useful for intervertebral disk degeneration evaluation. However, MRI is unavailable for patients with contraindicated metal implantation or claustrophobia. Moreover, MRI may not be an optimal tool for diagnosing other causative diseases of lower back pain than spinal diseases such as urinary tract stones, aortic dissection, etc. Computed tomography (CT) effectively evaluated these diseases, but its usefulness for lumbar intervertebral disk degeneration evaluation has not been established.

Spectral CT imaging encompasses a unique generation of CT systems that utilize energy-dependent information, with dual-energy CT (DECT) representing its first generation, which can be implemented through various technical approaches including dual-source CT, rapid kVp-switching, and dual-layer spectral detector CT (DLCT) [8]. DECT analyzes data of Compton scattering and the photoelectric effect of X–rays of two energies using various algorithms. DECT studies of intervertebral disk diseases have been reported in recent years, specifically including virtual non-calcium imaging for disk herniation and degeneration [9–13], water imaging for disk degeneration [14], collagen mapping for disk degeneration [15–17], and electron density (ED) imaging for disk herniation [12, 13]. ED and effective atomic number (Z_eff_) are recently available as DECT derived parameters [18]. DECT enable more accurate measurements of ED and Z_eff_ through improved dose distribution accuracy, which were difficult to obtain with conventional single-energy CT [19]. While these measurements have been primarily investigated for improving dose calculation accuracy in radiation treatment planning [20], their utility in diagnostic imaging has remained underexplored. Recently, however, the diagnostic value of ED for evaluating disk herniation has been reported [12, 13]. To the best of our knowledge, the use of ED and Z_eff_ images for lumbar intervertebral disk degeneration evaluation remains unknown. Therefore, this study aimed to investigate whether spectral CT-derived ED and Zeff can accurately differentiate varying degrees of lumbar disk degeneration. (P3, line4- P4, line12).

**[R#2-2] The term "DECT" is inaccurate here, as the authors did not employ "two energies" but4, lineead utilized a dual-layer detector. This terminology should be revised throughout the manuscript to reflect "spectral CT."**

We thank the reviewer for this comment. We have clarified the relationship between spectral CT, DECT, and DLCT in the Introduction and replaced "DECT" with "spectral CT" where appropriate throughout the manuscript.

New sentence: Spectral CT imaging encompasses a unique generation of CT systems that utilize energy-dependent information, with dual-energy CT (DECT) representing its first generation, which can be implemented through various technical approaches including dual-source CT, rapid kVp-switching, and dual-layer spectral detector CT (DLCT) [8]. (P4, line1-3).

**[R#2-3] Furthermore, scoring and reliability data for MRI are absent.**

We thank the reviewer for this comment. We have added the inter-observer agreement results for mPG (MRI-based disc degeneration grading) to the "Inter-observer agreements of mPG and spectral CT parameters" section in the Results.

New sentence: The inter-observer agreement for the mPG was substantial (κ = 0.80, 95% confidence interval [CI]：0.76-0.84). (P12, line2).

**[R#2-4] The authors should consider transferring some data from tables to figures (e.g., table 3) for better clarity.**

We thank the reviewer for this comment. We have changed Table 3 to Figure 5 as suggested. (P15, line9-13)

**[R#2-5] It is essential for the authors to complete a STARD checklist and provide the missing information, particularly the complete crosstabulation.**

We thank the reviewer for this comment. We have completed the STARD checklist and created the complete crosstabulation as requested. We provided completed checklist as a supplement. The complete crosstabulation has been included as Supplemental Table 2 in the Supporting Information (P27) and Supplemental Checklist 4. We added the following sentences in Material and Methods, Patient population.

New sentence: The institutional review board approved this retrospective study under protocol number 200284 and waived the need to obtain informed consent because of the retrospective study design. (P4, line4-P5, line1).

We used the STARD reporting guideline [21] to draft this manuscript, and the STARD reporting checklist [22] when editing, included in Supplemental Checklist 4 (P5, line8-9)

**[R#2-6] Lastly, the discussion lacks a comparison to existing literature.**

We thank the reviewer for this comment and added the following sentences in the Discussion to compare our findings with existing literature on intervertebral disc degeneration using VNCa and collagen mapping techniques.

New sentence: Previous studies have reported correlations between other spectral CT parameters, specifically VNC and collagen maps, and intervertebral disc degeneration. Shinohara et al. demonstrated that VNC values in NP showed a positive correlation with mPG (R² = 0.574, P < 0.05), whereas no significant correlation was observed between VNC values and mPG in the annulus fibrosus (AF) (R² = −0.015, P = 0.846) [10]. Pohlan et al reported that collagen map density in the anterior anulus fibrosus, but not in the nucleus pulposus, significantly declined with age (r = -0.09341, p = 0.0003), reflecting age-dependent reduction in collagen and proteoglycan content [15]. In this study, ED correlated with disc degeneration in both the NP and AF, and the difference in ED between these compartments showed a strong negative correlation with mPG. This contrasts with VNC, which correlated only with NP degeneration, and collagen maps, which reflected age-related changes only in the AF. Therefore, ED has the unique advantage of simultaneously assessing degenerative changes in both compartments of the intervertebral disc and may serve as a more comprehensive parameter for evaluating overall disc degeneration. (P19, line12-16-P20, line4).

Shinohara et al. reported that mean VNC values in the NP differed significantly between mPG grades (P < 0.05), except between grades 3 and 4 (P = 0.111), while mean VNC values in the AF showed no significant differences across mPG grades (P = 0.160–1.000) [13]. Regarding collagen maps, Bernatz et al. demonstrated an overall accuracy of 81% for visual assessment of disc degeneration [20]. While these spectral CT parameters, VNC and collagen maps, have proven useful in diagnosing disc degeneration, no studies to date, including the present investigation, have directly compared ED, VNC, and collagen maps in the context of disc degeneration. However, Jeong et al. directly compared ED, conventional CT, and VNC images for detecting lumbar disk herniation and reported that ED images achieved superior diagnostic performance due to lower coefficient of variation and higher normalized contrast ratio values [15]. This finding is consistent with our quantitative results demonstrating a stronger correlation between ED parameters and the degree of disk degeneration. Future studies should investigate whether comparative and integrated analyses of ED, VNC, and collagen mapping could further enhance diagnostic accuracy in the assessment of intervertebral disk degeneration. (P20, line10-16).

****Minor Points:****

**[R#2-7] I have concerns regarding the correlation analysis; it would be prudent to have it reviewed by a professional statistician.**

We thank the reviewer for this comment. All statistical analyses in this study, including the correlation analysis between mPG and spectral CT parameters, were consulted with Dr. Chihaya Koriyama, a professional statistician (Department of Epidemiology and Preventive Medicine, Kagoshima University Graduate School of Medical and Dental Sciences, Kagoshima, Japan).

Regarding the correlation analysis:

We employed Spearman's rank correlation coefficient because the parameters exhibited non-normal distributions, making a non-parametric approach appropriate. This analytical method is consistent with previous studies in this field (Shinohara Y, et al. Eur J Radiol 2020;124:108817). After consulting with Dr. Koriyama, we confirmed that this approach was statistically appropriate and no modification was necessary for the correlation analysis.

Regarding the inter-observer agreement analysis:

Based on your suggestion and consultation with Dr. Koriyama, we have revised our analytical approach. Initially, we used ICC for CT_conv_ and weighted kappa for ED and Zeff. We have revised the analysis to use ICC for all continuous variables (CT_conv_, ED, and Z_eff_).

Based on this consultation, we made minor revisions and additions to the statistical methods and Acknowledgements.

New sentences: The inter-observer agreement for the CT_conv_ was moderate (ICC = 0.56, 95% CI：0.52-0.59), whereas those for ED and Z_eff_ were almost perfect (ICC = 0.86, 95% CI：0.85-0.87), and substantial (ICC = 0.73, 95% CI：0.71-0.75), respectively. (P12,line2-4)

We thank Dr. Chihaya Koriyama, Department of Epidemiology and Preventive Medicine, Kagoshima University Graduate School of Medical and Dental Sciences, Kagoshima, Japan, for statistical review and advice. (P23, line1-2)

We hope that this extensively revised manuscript will meet with the reviewers’ approval.

Sincerely
