# Supplementary material for: Spectral CT electron density for evaluation of lumbar intervertebral disk degeneration
Source: PLoS One. 2026 Sep 1;21(9):e0357658. doi: 10.1371/journal.pone.0357658 (PMC13533366; doi:10.1371/journal.pone.0357658)
Supplement: Peer review history

# Peer review: Decision Letter 0

**DOI:** 10.1371/journal.pone.0357658.r001

**Authors:** Aloysius Gonzaga Mubuuke

29 Oct 2025

Dear Dr. Yoshiura,

Thank you for submitting your manuscript to PLOS ONE. After careful consideration, we feel that it has merit but does not fully meet PLOS ONE’s publication criteria as it currently stands. Therefore, we invite you to submit a revised version of the manuscript that addresses the points raised during the review process.

==============================

**ACADEMIC EDITOR: The introduction section needs to be more focused to the research problem and the research question should be clearly implied within your introduction. Second, you need to discuss the limitations around generalizability of your findings.**

Please submit your revised manuscript by Dec 13 2025 11:59PM. If you will need more time than this to complete your revisions, please reply to this message or contact the journal office at plosone@plos.org. When you're ready to submit your revision, log on to https://www.editorialmanager.com/pone/ and select the 'Submissions Needing Revision' folder to locate your manuscript file.

- A rebuttal letter that responds to each point raised by the academic editor and reviewer(s). You should upload this letter as a separate file labeled 'Response to Reviewers'.
- A marked-up copy of your manuscript that highlights changes made to the original version. You should upload this as a separate file labeled 'Revised Manuscript with Track Changes'.
- An unmarked version of your revised paper without tracked changes. You should upload this as a separate file labeled 'Manuscript'.

If you would like to make changes to your financial disclosure, please include your updated statement in your cover letter. Guidelines for resubmitting your figure files are available below the reviewer comments at the end of this letter.

If applicable, we recommend that you deposit your laboratory protocols in protocols.io to enhance the reproducibility of your results. Protocols.io assigns your protocol its own identifier (DOI) so that it can be cited independently in the future. For instructions see: https://journals.plos.org/plosone/s/submission-guidelines#loc-laboratory-protocols. Additionally, PLOS ONE offers an option for publishing peer-reviewed Lab Protocol articles, which describe protocols hosted on protocols.io. Read more information on sharing protocols at https://plos.org/protocols?utm_medium=editorial-email&utm_source=authorletters&utm_campaign=protocols.

We look forward to receiving your revised manuscript.

Kind regards,

Aloysius Gonzaga Mubuuke

Academic Editor

PLOS ONE

Journal requirements:

When submitting your revision, we need you to address these additional requirements.

1. Please ensure that your manuscript meets PLOS ONE's style requirements, including those for file naming. The PLOS ONE style templates can be found at

https://journals.plos.org/plosone/s/file?id=wjVg/PLOSOne_formatting_sample_main_body.pdf and

https://journals.plos.org/plosone/s/file?id=ba62/PLOSOne_formatting_sample_title_authors_affiliations.pdf

2. Thank you for stating the following financial disclosure:

[This study was supported by grants from the Grants-in-Aid for Scientific Research (KAKENHI) from the Japan Society for the Promotion of Science (JSPS) [Grant Number 21K15830].].

Please state what role the funders took in the study.  If the funders had no role, please state: "The funders had no role in study design, data collection and analysis, decision to publish, or preparation of the manuscript."

If this statement is not correct you must amend it as needed.

Please include this amended Role of Funder statement in your cover letter; we will change the online submission form on your behalf.

3. If the reviewer comments include a recommendation to cite specific previously published works, please review and evaluate these publications to determine whether they are relevant and should be cited. There is no requirement to cite these works unless the editor has indicated otherwise.

Additional Editor Comments (if provided):

[Note: HTML markup is below. Please do not edit.]

Reviewers' comments:

Reviewer's Responses to Questions

**Comments to the Author**

1. Is the manuscript technically sound, and do the data support the conclusions?

Reviewer #1: Yes

Reviewer #2: Partly

**********

2. Has the statistical analysis been performed appropriately and rigorously?

Reviewer #1: Yes

Reviewer #2: I Don't Know

**********

3. Have the authors made all data underlying the findings in their manuscript fully available?

The PLOS Data policy

Reviewer #1: Yes

Reviewer #2: No

**********

4. Is the manuscript presented in an intelligible fashion and written in standard English?

Reviewer #1: Yes

Reviewer #2: Yes

**********

Reviewer #1: I have carefully reviewed the manuscript and find it to be well written and scientifically sound. The objective is clearly stated, achievable, and the findings contribute meaningfully to the growing body of knowledge in computed tomography (CT) science. The study also highlights novel potential applications of CT technology. However, a few issues require clarification or further elaboration:

1. CT Dose Justification:

Did the researcher consider patient radiation dose during CT examinations for intervertebral degeneration assessment? Please clarify whether the use of CT for this indication is justified in terms of benefit versus exposure.

2. Introduction Contextual Clarity:

The introduction mentions that DECT studies of intervertebral disk diseases have been reported in recent years. Although this statement is referenced, it would strengthen the background if specific examples of such disk diseases were mentioned. Additionally, the author should explain why effective atomic number (Zeff) and electron density (ED) images for lumbar intervertebral disk degeneration (LIDD) remain underexplored. Are there particular research or technical limitations contributing to this gap?

3. Methods Reader Experience:

Under the analysis section, there appears to be a considerable difference in professional experience between the two radiologists (8 years vs. 17 years). Please clarify whether both radiologists possess similar expertise in DECT interpretation and whether this variation could have influenced the image reading accuracy or overall results.

4. Generalisability:

The study appears to have been conducted at a single center. The authors should discuss how this limitation might affect the generalisability of the findings to other clinical settings or populations.

5. Clinical Applicability:

Based on the study outcomes, do the authors recommend adopting this DECT-based method for clinical assessment of intervertebral disk degeneration, or is further validation and research still warranted before routine clinical implementation?

Overall, this is a well-conceived and informative paper, and with the above clarifications, it could make a valuable contribution to the field.

Reviewer #2: The authors present a study on spectral CT for assessing lumbar disc degeneration, utilizing basic parameters such as electron density (ED) and effective atomic number (Zeff) to characterize the intervertebral disc. However, they overlook a comparison or discussion of previously reported techniques, such as VNCa and collagen mapping. The results and discussion sections are lengthy and lack organization. Additionally, the authors miss the opportunity to clearly articulate the hypothesis of their work in the introduction. Below are further specific points for consideration.

**Major Points:**

The introduction is overly broad and lacks specificity, reiterating established facts. I recommend streamlining it to focus on spectral CT and discussing why the authors believe electron density is a valuable target, especially since this approach has not been previously explored in depth.

The term "DECT" is inaccurate here, as the authors did not employ "two energies" but instead utilized a dual-layer detector. This terminology should be revised throughout the manuscript to reflect "spectral CT."

Furthermore, scoring and reliability data for MRI are absent.

The authors should consider transferring some data from tables to figures (e.g., table 3) for better clarity.

It is essential for the authors to complete a STARD checklist and provide the missing information, particularly the complete crosstabulation.

Lastly, the discussion lacks a comparison to existing literature.

**Minor Points:**

I have concerns regarding the correlation analysis; it would be prudent to have it reviewed by a professional statistician.

**********

what does this mean?). If published, this will include your full peer review and any attached files.

If you choose “no”, your identity will remain anonymous but your review may still be made public.

**Do you want your identity to be public for this peer review?**  For information about this choice, including consent withdrawal, please see our Privacy Policy

Reviewer #1: **Yes:** Mr. Alen Musisi

Reviewer #2: No

**********

[NOTE: If reviewer comments were submitted as an attachment file, they will be attached to this email and accessible via the submission site. Please log into your account, locate the manuscript record, and check for the action link "View Attachments". If this link does not appear, there are no attachment files.]

To ensure your figures meet our technical requirements, please review our figure guidelines: https://journals.plos.org/plosone/s/figures

You may also use PLOS’s free figure tool, NAAS, to help you prepare publication quality figures: https://journals.plos.org/plosone/s/figures#loc-tools-for-figure-preparation.

NAAS will assess whether your figures meet our technical requirements by comparing each figure against our figure specifications.

# Author comment: Author response to Decision Letter 1

**DOI:** 10.1371/journal.pone.0357658.r002

26 Jan 2026

EDITOR’S COMMENTS:

[E# 1] The introduction section needs to be more focused to the research problem and the research question should be clearly implied within your introduction.

We thank the editor for this comment and have deleted some sentences to focus on spectral CT and research problem.

New sentence: Lumbar intervertebral disk degeneration is the most prevalent cause of low back pain [1, 2], and it causes herniated disk disease, lumbar degenerative spondylosis, and lumbar spinal canal stenosis [3].

The intervertebral disk is centrally and peripherally composed of the nucleus pulposus (NP) and the annulus fibrosus (AF), respectively. The AF consists of a fibrocartilaginous structure that primarily contains type I collagen, whereas the NP is rich in water and proteoglycans in a loose network of type II collagen [4]. Proteoglycans in disk degeneration, which function to bind water, decrease, thereby causing dehydration and relatively increased type I collagen within the NP, and tears and degenerate cysts of AF [5]. Lumbar intervertebral disk degeneration evaluation is conventionally performed using Magnetic resonance imaging (MRI) [1, 2, 5–7]. Pfirrmann grade and modified Pfirrmann grade (mPG) based on T2-weighted images are predominantly used for grading degenerative disk disease [6, 7]. Thus, MRI is useful for intervertebral disk degeneration evaluation. However, MRI is unavailable for patients with contraindicated metal implantation or claustrophobia. Moreover, MRI may not be an optimal tool for diagnosing other causative diseases of lower back pain than spinal diseases such as urinary tract stones, aortic dissection, etc. Computed tomography (CT) effectively evaluated these diseases, but its usefulness for lumbar intervertebral disk degeneration evaluation has not been established.

Spectral CT imaging encompasses a unique generation of CT systems that utilize energy-dependent information, with dual-energy CT (DECT) representing its first generation, which can be implemented through various technical approaches including dual-source CT, rapid kVp-switching, and dual-layer spectral detector CT (DLCT) [8]. DECT analyzes data of Compton scattering and the photoelectric effect of X–rays of two energies using various algorithms. DECT studies of intervertebral disk diseases have been reported in recent years, specifically including virtual non-calcium imaging for disk herniation and degeneration [9–13], water imaging for disk degeneration [14], collagen mapping for disk degeneration [15–17], and electron density (ED) imaging for disk herniation [12, 13]. ED and effective atomic number (Zeff) are recently available as DECT derived parameters [18]. DECT enable more accurate measurements of ED and Zeff through improved dose distribution accuracy, which were difficult to obtain with conventional single-energy CT [19]. While these measurements have been primarily investigated for improving dose calculation accuracy in radiation treatment planning [20], their utility in diagnostic imaging has remained underexplored. Recently, however, the diagnostic value of ED for evaluating disk herniation has been reported [12, 13]. To the best of our knowledge, the use of ED and Zeff images for lumbar intervertebral disk degeneration evaluation remains unknown. Therefore, this study aimed to investigate whether spectral CT-derived ED and Zeff can accurately differentiate varying degrees of lumbar disk degeneration. (P3, line4- P4, line12).

[E# 2]Second, you need to discuss the limitations around generalizability of your findings.

We thank the editor for this comment and added the following sentences as a limitation in Discussion according to Editor’s suggestion.

New sentence: First, this was a single-center retrospective study involving patients with suspected spinal and spinal cord diseases, without healthy controls. The use of a single spectral CT platform (Philips Healthcare DLCT) may limit generalizability, and selection bias related to disease characteristics and severity cannot be excluded. (P21,line13-15).

REVIEWER’S COMMENTS

Reviewer# 1

I have carefully reviewed the manuscript and find it to be well written and scientifically sound. The objective is clearly stated, achievable, and the findings contribute meaningfully to the growing body of knowledge in computed tomography (CT) science. The study also highlights novel potential applications of CT technology. However, a few issues require clarification or further elaboration:

[R# 1-1] 1. CT Dose Justification:

Did the researcher consider patient radiation dose during CT examinations for intervertebral degeneration assessment? Please clarify whether the use of CT for this indication is justified in terms of benefit versus exposure.

We thank the reviewer for this comment and added the following sentences as a limitation in Discussion according to reviewer’s suggestion.

New sentence: Fourth, the diagnostic performance of spectral CT did not reach the level of MRI, which remains the gold standard for disk degeneration assessment. Given the radiation exposure, our findings do not justify routine use of CT for disk degeneration assessment. At present, spectral CT may serve as an alternative imaging modality for patients with MRI contraindications (e.g., pacemakers, claustrophobia, metallic implants) or when simultaneous evaluation of other causes of low back pain is needed. Future multicenter prospective studies involving multiple spectral CT platforms from different vendors are warranted to validate inter-vendor reproducibility and establish the clinical utility of spectral CT parameters across diverse patient populations and technologies. (P22, line4-10).

[R# 1-2] 2. Introduction Contextual Clarity:

The introduction mentions that DECT studies of intervertebral disk diseases have been reported in recent years. Although this statement is referenced, it would strengthen the background if specific examples of such disk diseases were mentioned. Additionally, the author should explain why effective atomic number (Zeff) and electron density (ED) images for lumbar intervertebral disk degeneration (LIDD) remain underexplored. Are there particular research or technical limitations contributing to this gap?

We thank the reviewer for this comment and added the following sentences in Introduction according to reviewer’s suggestion.

New sentence: DECT studies of intervertebral disk diseases have been reported in recent years, specifically including virtual non-calcium imaging for disk herniation and degeneration [9–13], water imaging for disk degeneration [14], collagen mapping for disk degeneration [15–17], and electron density (ED) imaging for disk herniation [12, 13]. ED and effective atomic number (Zeff) are recently available as DECT derived parameters [18]. DECT enable more accurate measurements of ED and Zeff through improved dose distribution accuracy, which were difficult to obtain with conventional single-energy CT [19]. While these measurements have been primarily investigated for improving dose calculation accuracy in radiation treatment planning [20], their utility in diagnostic imaging has remained underexplored. Recently, however, the diagnostic value of ED for evaluating disk herniation has been reported [12, 13] (P4, line4-10).

[R# 1-3]3. Methods Reader Experience:

Under the analysis section, there appears to be a considerable difference in professional experience between the two radiologists (8 years vs. 17 years). Please clarify whether both radiologists possess similar expertise in DECT interpretation and whether this variation could have influenced the image reading accuracy or overall results.

We thank the reviewer for this comment and added the following sentences as a limitation in Discussion according to reviewer’s suggestion.

New sentence: Third, inter-observer agreement for ROI measurements was moderate to substantial (mPG: weighted κ = 0.80; ED: ICC = 0.86; Zeff: ICC = 0.73; CTconv: ICC = 0.56), indicating suboptimal reproducibility. This may be attributed to manual ROI placement on single midsagittal images; volumetric measurements might improve reproducibility and accuracy. The lower agreement for CTconv likely reflects inherent variability in conventional CT measurements rather than reader-related factors such as differences in radiological experience, as identical ROIs were used across all parameter maps. (P21, line16-P22, line4).

[R# 1-4]4. Generalisability:

The study appears to have been conducted at a single center. The authors should discuss how this limitation might affect the generalisability of the findings to other clinical settings or populations.

We thank the reviewer for this comment and added the following sentences as a limitation in Discussion according to reviewer’s suggestion.

New sentence: First, this was a single-center retrospective study involving patients with suspected spinal and spinal cord diseases, without healthy controls. The use of a single spectral CT platform (Philips Healthcare DLCT) may limit generalizability, and selection bias related to disease characteristics and severity cannot be excluded. (P21,line13-15).

[R# 1-5]5. Clinical Applicability:

Based on the study outcomes, do the authors recommend adopting this DECT-based method for clinical assessment of intervertebral disk degeneration, or is further validation and research still warranted before routine clinical implementation?

Overall, this is a well-conceived and informative paper, and with the above clarifications, it could make a valuable contribution to the field.

We thank the reviewer for this comment and added the following sentences as a limitation in Discussion according to reviewer’s suggestion.

New sentence: Fourth, the diagnostic performance of spectral CT did not reach the level of MRI, which remains the gold standard for disk degeneration assessment. Given the radiation exposure, our findings do not justify routine use of CT for disk degeneration assessment. At present, spectral CT may serve as an alternative imaging modality for patients with MRI contraindications (e.g., pacemakers, claustrophobia, metallic implants) or when simultaneous evaluation of other causes of low back pain is needed. Future multicenter prospective studies involving multiple spectral CT platforms from different vendors are warranted to validate inter-vendor reproducibility and establish the clinical utility of spectral CT parameters across diverse patient populations and technologies. (P21,line4-10).

Reviewer #2:

The authors present a study on spectral CT for assessing lumbar disc degeneration, utilizing basic parameters such as electron density (ED) and effective atomic number (Zeff) to characterize the intervertebral disc. However, they overlook a comparison or discussion of previously reported techniques, such as VNCa and collagen mapping. The results and discussion sections are lengthy and lack organization. Additionally, the authors miss the opportunity to clearly articulate the hypothesis of their work in the introduction. Below are further specific points for consideration.

**Major Points:**

[R#2-1] The introduction is overly broad and lacks specificity, reiterating established facts. I recommend streamlining it to focus on spectral CT and discussing why the authors believe electron density is a valuable target, especially since this approach has not been previously explored in depth.

We thank the reviewer for this comment and have deleted some sentences to focus on spectral CT and research problem.

New sentence: Lumbar intervertebral disk degeneration is the most prevalent cause of low back pain [1, 2], and it causes herniated disk disease, lumbar degenerative spondylosis, and lumbar spinal canal stenosis [3].

The intervertebral disk is centrally and peripherally composed of the nucleus pulposus (NP) and the annulus fibrosus (AF), respectively. The AF consists of a fibrocartilaginous structure that primarily contains type I collagen, whereas the NP is rich in water and proteoglycans in a loose network of type II collagen [4]. Proteoglycans in disk degeneration, which function to bind water, decrease, thereby causing dehydration and relatively increased type I collagen within the NP, and tears and degenerate cysts of AF [5]. Lumbar intervertebral disk degeneration evaluation is conventionally performed using Magnetic resonance imaging (MRI) [1, 2, 5–7]. Pfirrmann grade and modified Pfirrmann grade (mPG) based on T2-weighted images are predominantly used for grading degenerative disk disease [6, 7]. Thus, MRI is useful for intervertebral disk degeneration evaluation. However, MRI is unavailable for patients with contraindicated metal implantation or claustrophobia. Moreover, MRI may not be an optimal tool for diagnosing other causative diseases of lower back pain than spinal diseases such as urinary tract stones, aortic dissection, etc. Computed tomography (CT) effectively evaluated these diseases, but its usefulness for lumbar intervertebral disk degeneration evaluation has not been established.

Spectral CT imaging encompasses a unique generation of CT systems that utilize energy-dependent information, with dual-energy CT (DECT) representing its first generation, which can be implemented through various technical approaches including dual-source CT, rapid kVp-switching, and dual-layer spectral detector CT (DLCT) [8]. DECT analyzes data of Compton scattering and the photoelectric effect of X–rays of two energies using various algorithms. DECT studies of intervertebral disk diseases have been reported in recent years, specifically including virtual non-calcium imaging for disk herniation and degeneration [9–13], water imaging for disk degeneration [14], collagen mapping for disk degeneration [15–17], and electron density (ED) imaging for disk herniation [12, 13]. ED and effective atomic number (Zeff) are recently available as DECT derived parameters [18]. DECT enable more accurate measurements of ED and Zeff through improved dose distribution accuracy, which were difficult to obtain with conventional single-energy CT [19]. While these measurements have been primarily investigated for improving dose calculation accuracy in radiation treatment planning [20], their utility in diagnostic imaging has remained underexplored. Recently, however, the diagnostic value of ED for evaluating disk herniation has been reported [12, 13]. To the best of our knowledge, the use of ED and Zeff images for lumbar intervertebral disk degeneration evaluation remains unknown. Therefore, this study aimed to investigate whether spectral CT-derived ED and Zeff can accurately differentiate varying degrees of lumbar disk degeneration. (P3, line4- P4, line12).

[R#2-2] The term "DECT" is inaccurate here, as the authors did not employ "two energies" but4, lineead utilized a dual-layer detector. This terminology should be revised throughout the manuscript to reflect "spectral CT."

We thank the reviewer for this comment. We have clarified the relationship between spectral CT, DECT, and DLCT in the Introduction and replaced "DECT" with "spectral CT" where appropriate throughout the manuscript.

New sentence: Spectral CT imaging encompasses a unique generation of CT systems that utilize energy-dependent information, with dual-energy CT (DECT) representing its first generation, which can be implemented through various technical approaches including dual-source CT, rapid kVp-switching, and dual-layer spectral detector CT (DLCT) [8]. (P4, line1-3).

[R#2-3] Furthermore, scoring and reliability data for MRI are absent.

We thank the reviewer for this comment. We have added the inter-observer agreement results for mPG (MRI-based disc degeneration grading) to the "Inter-observer agreements of mPG and spectral CT parameters" section in the Results.

New sentence: The inter-observer agreement for the mPG was substantial (κ = 0.80, 95% confidence interval [CI]：0.76-0.84). (P12, line2).

[R#2-4] The authors should consider transferring some data from tables to figures (e.g., table 3) for better clarity.

We thank the reviewer for this comment. We have changed Table 3 to Figure 5 as suggested. (P15, line9-13)

[R#2-5] It is essential for the authors to complete a STARD checklist and pr

Attachment Submitted filename: Response to Reviewers.doc

# Peer review: Decision Letter 1

**DOI:** 10.1371/journal.pone.0357658.r003

**Authors:** Ali Fathi Jouzdani

20 Aug 2026

Spectral CT electron density for evaluation of lumbar intervertebral disk degeneration

PONE-D-25-20109R1

Dear Dr. Yoshiura,

We’re pleased to inform you that your manuscript has been judged scientifically suitable for publication and will be formally accepted for publication once it meets all outstanding technical requirements.

Within one week, you’ll receive an e-mail detailing the required amendments. When these have been addressed, you’ll receive a formal acceptance letter and your manuscript will be scheduled for publication.

An invoice will be generated when your article is formally accepted. Please note, if your institution has a publishing partnership with PLOS and your article meets the relevant criteria, all or part of your publication costs will be covered. Please make sure your user information is up-to-date by logging into Editorial Manager at Editorial Manager® and clicking the ‘Update My Information' link at the top of the page. For questions related to billing, please contact billing support.

If your institution or institutions have a press office, please notify them about your upcoming paper to help maximize its impact. If they’ll be preparing press materials, please inform our press team as soon as possible -- no later than 48 hours after receiving the formal acceptance. Your manuscript will remain under strict press embargo until 2 pm Eastern Time on the date of publication. For more information, please contact onepress@plos.org.

Kind regards,

Ali Fathi Jouzdani, M.D.

Guest Editor

PLOS One

Additional Editor Comments (optional):

Reviewers' comments:

Reviewer's Responses to Questions

**Comments to the Author**

Reviewer #2: All comments have been addressed

**********

2. Is the manuscript technically sound, and do the data support the conclusions?

Reviewer #2: Yes

**********

3. Has the statistical analysis been performed appropriately and rigorously?

Reviewer #2: Yes

**********

4. Have the authors made all data underlying the findings in their manuscript fully available?

The PLOS Data policy

Reviewer #2: Yes

**********

5. Is the manuscript presented in an intelligible fashion and written in standard English?

Reviewer #2: Yes

**********

Reviewer #2: Thank you for your thorough revisions and for addressing all of my previously raised issues and comments.

**********

what does this mean?). If published, this will include your full peer review and any attached files.

If you choose “no”, your identity will remain anonymous but your review may still be made public.

**Do you want your identity to be public for this peer review?**  For information about this choice, including consent withdrawal, please see our Privacy Policy

Reviewer #2: No

**********

# Editor report: Acceptance letter

**DOI:** 10.1371/journal.pone.0357658.r004

**Authors:** Ali Fathi Jouzdani

PONE-D-25-20109R1

PLOS One

Dear Dr. Yoshiura,

I'm pleased to inform you that your manuscript has been deemed suitable for publication in PLOS One. Congratulations! Your manuscript is now being handed over to our production team.

At this stage, our production department will prepare your paper for publication. This includes ensuring the following:

* All references, tables, and figures are properly cited

* All relevant supporting information is included in the manuscript submission,

* There are no issues that prevent the paper from being properly typeset

You will receive further instructions from the production team, including instructions on how to review your proof when it is ready. Please keep in mind that we are working through a large volume of accepted articles, so please give us a few days to review your paper and let you know the next and final steps.

Lastly, if your institution or institutions have a press office, please let them know about your upcoming paper now to help maximize its impact. If they'll be preparing press materials, please inform our press team within the next 48 hours. Your manuscript will remain under strict press embargo until 2 pm Eastern Time on the date of publication. For more information, please contact onepress@plos.org.

You will receive an invoice from PLOS for your publication fee after your manuscript has reached the completed accept phase. If you receive an email requesting payment before acceptance or for any other service, this may be a phishing scheme. Learn how to identify phishing emails and protect your accounts at https://explore.plos.org/phishing.

If we can help with anything else, please email us at customercare@plos.org.

Thank you for submitting your work to PLOS One and supporting open access.

Kind regards,

PLOS One Editorial Office Staff

on behalf of

Dr. Ali Fathi Jouzdani

Guest Editor

PLOS One
